# Halophytic Grasses, a New Source of Nutraceuticals? A Review on Their Secondary Metabolites and Biological Activities

**DOI:** 10.3390/ijms20051067

**Published:** 2019-03-01

**Authors:** Maria V. Faustino, Maria A. F. Faustino, Diana C. G. A. Pinto

**Affiliations:** QOPNA & LAQV-REQUIMTE, Department of Chemistry, University of Aveiro, 3810-193 Aveiro, Portugal; maria.vf9@ua.pt (M.V.F.); faustino@ua.pt (M.A.F.F.)

**Keywords:** Poaceae, halophytes, phytoconstituents, bioactivity, nutraceuticals

## Abstract

The Poaceae family, known as grasses, is distributed worldwide and is considered the most important group of monocotyledonous crops. Salt stress is multifactorial, therefore to survive, halophytes evolved a variety of adaptations, which include the biosynthesis of different primary and secondary metabolites. This trait enhances the accumulation of important families of compounds crucial to the prevention of a variety of chronic diseases. Besides, if proven edible, these species could cope with the increased soil salinity responsible for the decline of arable land due to their high nutritional/nutraceutical value. Herein, the phytochemical investigations performed in halophytes from the Poaceae family as well as their biological properties were explored. Among the 65 genera and 148 species of known halophytic grasses, only 14% of the taxa were studied phytochemically and 10% were subjected to biological evaluation. Notably, in the studied species, a variety of compound families, as well as bioactivities, were demonstrated, highlighting the potential of halophytic grasses.

## 1. Introduction

The Poaceae is a large family of monocotyledonous plants, commonly recognized as grasses, representing the most important group of crops [1]. This family encompasses several noteworthy cultivated species such as *Triticum aestivum* L., *Oryza sativa* L., *Zea mays* L. and *Hordeum vulgare* L. [2]. It comprises of 7500 species [3] distributed worldwide and with a wide spectrum of climatic adaptations. Remarkably, grasses also show extreme ranges in salinity tolerance, from salt sensitive (glycophytic) to extremely tolerant (halophytic) [4]. For instance, *Poa annua* L. is highly sensitive, *Paspalum dilatatum* Poir. is moderately salt tolerant and *Cynodon dactylon* (L.) Pers. is completely tolerant (true halophyte) [4]. A completely salt tolerant species is defined by the ability to complete a life cycle in a salt concentration of at least 200 mM of NaCl [5]. Nowadays, 1560 species from 550 genera and 117 families [6] are known to have salt-tolerance, among these the Poaceae family includes 65 genera and 148 species [7] of halophytes.

Halophytes can keep and acquire water, protect cells from the damage caused by the accumulation of reactive oxygen species (ROS), and maintain ion homeostasis in salty stressed environments through a variety of adaptations [5]. These include the biosynthesis of different biocompounds that can be useful due to their biological activities, such as antioxidant, antimicrobial, anti-inflammatory, and antitumoral [8]. In this vein, they can be crucial for the prevention of a variety of diseases as, for instance, cancer, chronic inflammation, and cardiovascular disorders when introduced in the human diet [9]. These compounds also enhance the nutraceutical value of halophytic grasses, since their concentration and/or diversity is increased when compared to no salt-tolerant crop species [10]. Notably, some molecules are even restricted to halophyte species and present high potential for its use in agri-food, pharmaceutical and cosmetic industries [11].

Due to their important ecological role related to the ability to cope with high concentrations of toxic ions as well as to the capacity to accumulate heavy metals present in the environment [12], studies have been dedicated to their potential as stabilizers and/or phytoextractors of heavy metal polluted soils [12,13,14,15,16,17,18,19,20,21,22,23,24,25,26,27,28,29,30,31,32,33,34,35,36,37,38,39,40]. Nonetheless, their phytochemistry and biological activities have not been equally well investigated. Taking into consideration the small number of published studies regarding these topics and the high diversity of halophytic grasses, these species represent an almost unexplored pool of novel bioactive compounds as well as a novel source for known compounds [41]. On top of this, several halophytic grasses such as *Beckmannia syzigachne* (Steud.) Fernald [42], *Arundo donax* L. [43], *Desmostachya bipinnata* (L.) Stapf [44], *Cenchrus ciliaris* L. [45], among several others, have been used in traditional medicine. Such practices also suggest that these halophytic grasses might contain chemical constituents with broad biological activities.

In addition, halophytic grasses could be a reservoir of edible and highly nutritional plants which is vital today, since in the past decade, the world population has increased continuously while a constant reduction of arable lands is observed due to increased soil salinity [46]. It is estimated that 1000 million hectares of land are affected by this issue, which corresponds to 20% of the world-cultivated area [47]. Soil salinity is considered a serious threat to global food security and sustainability [46]; however, a glimmer of hope lies on the existence of some truly salt-tolerant plants from Poaceae, which can survive in seawater salt concentrations and simultaneously have high nutraceutical potential [48]. For instance, some of these species have already been considered edible for cattle, which is the case of *Beckmannia syzigachne* (Steud.) Fernald [49], *Cenchrus ciliaris* L. [50], *Echinochloa colona* (L.) Link [51], *Echinochloa crus-galli* (L.) P.Beauv [51], *Dactyloctenium aegyptium* (L.) Willd. [52], *Imperata cylindrica* (L.) Raeusch. [53], *Leymus arenarius* (L.) Hochst. [54], *Phragmites australis* (Cav.) Trin. ex Steud. [49] and *Zizania aquatica* L. [55].

Halophytic grasses represent a possible solution to solve the agriculture crises related to the increased soil salinity [10], as well as a new source of bioactive compounds that could be exploited by pharmaceutical and cosmetic industries [8]. In this review, we focus on the chemical profile and biological activities of halophytes from the Poaceae, aiming to clarify, for the first time, their nutritional and medicinal potential as well as their value as a source of new drugs. The conducted literature review was achieved by using the database Scopus and PubMed.

## 2. Phytoconstituents of Halophytic Grasses

**Figure 1:**
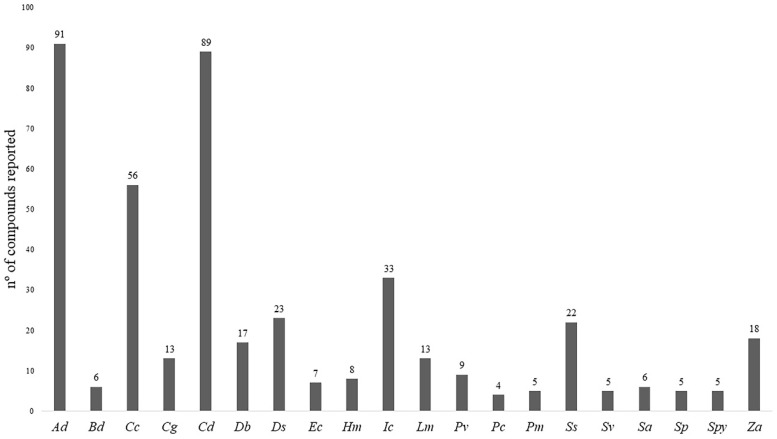
Total number of compounds reported in each species of halophytic grasses. Being, ***Ad***: *Arundo donax; **Bd***: *Buchloe dactyloides*; ***Cc*:** *Cenchrus ciliaris*; **Cg:** *Chloris gayana*; ***Cd*:** *Cynodon dactylon*; ***Db***: Desmostachya bipinnata; ***Ds***: *Distichlis spicata*; ***Ec***: *Echinochloa crus-galli; **Hm*****:** *Halopyrum mucronatum*; ***Im*:** *Imperata cylindrica*; ***Lm***: *Lolium multiflorum*; ***Pv***: *Panicum virgatum*; ***Pc***: *Pennisetum clandestinum*; ***Pm***: *Puccinellia maritima*; ***Ss***: *Saccharum spontaneum*; ***Sv***: *Setaria viridis*; ***Sa***: *Spartina anglica*; ***Sp***: *Spartina patens*; ***Spy***: *Sporobolus pyramidalis* and ***Za***: *Zizania aquatica*.

Of the 148 species distributed in 65 genera [7], only 20 were studied regarding this aspect, summing 14% of the diversity of the *taxa* (Table S1 and Figure 1). These include *Arundo donax* L., *Buchloe dactyloides* (Nutt.) Engelm., *Cenchrus ciliaris* L., *Chloris gayana* Kunth, *Cynodon dactylon* (L.) Pers., *Desmostachya bipinnata* (L.) Stapf, *Distichlis spicata* (L.) Greene, *Echinochloa crus-galli* (L.) P. Beauv., *Halopyrum mucronatum* (L.) Stapf, *Imperata cylindrica* (L.) Raeusch, *Lolium multiflorum* Lam., *Panicum virgatum* L., *Pennisetum clandestinum* Hochst. ex Chiov., *Puccinellia maritima* (Huds.) Parl., *Saccharum spontaneum* L., *Setaria viridis* (L.) P. Beauv., *Spartina anglica* C. E. Hubb., *Spartina patens* (Aiton.) Muhl., *Sporobolus pyramidalis* P. Beauv. and *Zizania aquatica* L. (Table S1). Among these, the most studied one was *A. donax*, with 92 compounds reported, followed by *C. dactylon*, with 82 and *C. ciliaris*, with 56. For the remaining species, between 4 and 33 compounds were described in the literature (Figure 1).

**Figure 2:**
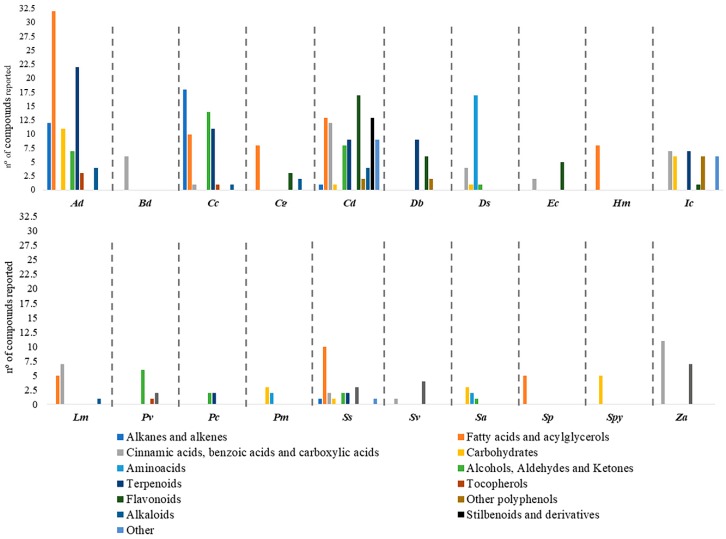
Graphical presentation of the total number of compounds reported in each class of compounds for the halophytic grasses studied. Being, ***Ad***: *Arundo donax; **Bd***: *Buchloe dactyloides*; ***Cc*:** *Cenchrus ciliaris*; **Cg:** *Chloris gayana*; ***Cd*:** *Cynodon dactylon*; ***Db***: Desmostachya bipinnata; ***Ds***: *Distichlis spicata*; ***Ec***: *Echinochloa crus-galli; **Hm*****:** *Halopyrum mucronatum*; ***Im*:** *Imperata cylindrica*; ***Lm***: *Lolium multiflorum*; ***Pv***: *Panicum virgatum*; ***Pc***: *Pennisetum clandestinum*; ***Pm***: *Puccinellia maritima*; ***Ss***: *Saccharum spontaneum*; ***Sv***: *Setaria viridis*; ***Sa***: *Spartina anglica*; ***Sp***: *Spartina patens*; ***Spy***: *Sporobolus pyramidalis* and ***Za***: *Zizania aquatica*.

Studies have reported the presence of alkanes and alkenes, fatty acids and acylglycerols, cinnamic acids, benzoic acids, short chain carboxylic acids, carbohydrates, amino acids, alcohols, aldehydes, ketones, terpenoids, tocopherols, flavonoids as well as other polyphenols, alkaloids, stilbenoids and derivatives and other miscellaneous compounds (Table S1). Regarding the diversity of classes reported in the different species, *C. dactylon* has 11 classes, followed by *S. spontaneum* with 8, *A. donax* and *C. ciliaris* with 7, and *I. cylindrica* with 6, the remaining species have between 1 and 4 classes of compounds reported (Figure 2). This puts in evidence the lack of research in some of the species discussed in this review. For instance, in *P. clandestinum*, only 4 compounds were described although distributed in 2 classes (Table S1). In spite of this, the phytochemical investigations have revealed that many compounds are highly bioactive. The complete description and distribution of the compounds reported in each species are illustrated in Table S1. Some chemical families such as alcohols, aldehydes and ketonesare not going to be explored in the text due to its lack of relevance from a medicinal and/or nutritional point of view. Others, such as tocopherols and alkaloids, will not be explored due to the low number of compounds described in halophytes from the Poaceae.

### 2.1. Alkanes and Alkenes

Alkanes and alkenes (compounds **1**–**29**, Table S1) were reported in *A. donax*, *C. ciliaris*, *C. dactylon*, and *S. spontaneum* species. In general, 29 molecules from this class were described: 18 in *C. ciliaris* [56,57], 12 in *A. donax* [43] and only 1 in both *C. dactylon* [58] and *S. spontaneum* [59] (Table S1). In the first species, these were obtained from its essential oils [57] as well as from the lipophilic, methanol and ethyl acetate root extracts [56] while in *A. donax* the alkanes were obtained from the lipophilic extracts [43]. 

Alkanes represent between 70% to 80% of the wax cuticle constitution, which is indispensable to prevent uncontrolled water loss [60]. Therefore, their role in abiotic stresses such as salinity stress is crucial [61]. The well-reported presence of these compounds in *C. ciliaris* and *A. donax* can be a sign of their adaptation to high salinity environments. Consequently, more studies must be conducted in order to completely characterize the presence of alkanes in other halophytic species. Some of the compounds included in this class are biologically active; for instance, tetradecane (**5**) [56], hexadecane (**7**) [56], heptadecane (**8**) [56], nonadecane (**10**) [56] and eicosane (**11**) [56] exhibited antimicrobial activities. In addition, nonacosane (**20**) seems to inhibit human gastric cancer cells BGC823 at 5 µM after 72 hours [62]. It was also found that hentriacontane (**22**) significantly reduced all the parameters of inflammation in the conducted experiments at all the tested concentrations: 10 μM, 5 μM and 1 μM (in vitro) and 5 mg/kg, 2 mg/kg and 1 mg/kg (in vivo) [63]. This emphasis the urge for more studies to understand alkanes’ role in salinity stress, but also the establishment of the alkane profiles of other Poaceaespecies.

### 2.2. Fatty Acids, Acylglycerols And Derivatives

Fatty acids are the second most described class of compounds in halophytic grasses, with their presence reported in eight species: *A. donax*, *C. ciliaris*, *C. gayana*, *C. dactylon*, *H. mucronatum*, *L. multiflorum*, *S. spontaneum* and *S. patens* (Table S1 and Figure 2). *A. donax* has the most detailed description on fatty acid composition with 21 compounds reported on its lipophilic extract [43]. Furthermore, 10 compounds were retrieved from the methanol, ethyl acetate, hexane root extract [56] and aerial parts essential oil [57] of *C. ciliaris*. In the case of *C. dactylon*, 13 compounds were obtained from the surface cuticular wax [58], hydroalcoholic extract [64,65] and ethanol extract (80%) [66] (Table S1). In *H. mucronatum*, from methanol/chloroform (1:2 *v*/*v*) extract [67], eight compounds were reported while in *L. multiflorum* only five were described (Table S1). At last, 10 fatty acids were retrieved from carbon tetrachloride extract (It should be emphasized that the use of carbon tetrachloride is not recommended due to its toxicity.) [59] of *S. spontaneum*, eight from the hexane extract of *C. gayana* [68] and five by direct acidic trans-esterification from *S. patens* [69].

Fatty acids reported in halophytes from the Poaceae comprised of saturated mid-chain and long chain fatty acids and unsaturated long chain fatty acids. Among these, three unsaturated and five polyunsaturated fatty acids (PUFAs) were reported (Table S1). These compounds play key roles in human physiology as they are building blocks of phospholipids and glycolipids [70]. Additionally, they also change proteins by covalent attachment, which targets them to membrane locations and their derivatives serve as hormones and intracellular messengers [70]. Palmitic acid (**41**), a saturated fatty acid, reported in *A. donax*, *C. ciliaris*, *C. dactylon*, *C. gayana*, *H. mucronatum*, and *S. patens*, is known for its ability to increase blood HDL-cholesterol levels without changes in the overall cholesterol/HDL-cholesterol ratio [71]. Its antioxidant, hemolytic and anticarcinogenic activities were also described [56].

Polyunsaturated fatty acids (PUFAs) contribute greatly to the resistance to photoinhibition of halophyte species, with their concentration increased in membrane lipids, which enhances the tolerance of photosystem II to salt stress [72]. This could explain the wide distribution of these compounds among halophytic grasses (Table S1 and Figure 2). PUFAs are also gaining attention of the scientific community due to their broad pharmacological properties [73]. An important aspect of these compounds is the direct link between dietary PUFAs’ content and lower blood cholesterol levels [74], which is observed for instance with linolenic acid (**52**) [75]. In addition, the antioxidant, anti-inflammatory, anti-osteoporosis, anticarcinogenic, neuroprotective and cardioprotective properties of linolenic acid have also been studied [56]. At the same time, linoleic acid (**50**), another polyunsaturated fatty acid, is involved in the synthesis of prostaglandins, thromboxanes and leukotriene, a fact recognized over the last century [76]. Several studies have also reported its antimicrobial [77] and anti-inflammatory activities [78].

Regarding acylglycerols, these were only described in the aqueous extract of *A. donax* [43]; in total, 11 compounds were reported (Table S1). Acylglycerols serve as storage of lipids and are of great nutritional value, being a common source of edible oils for alimentation and industrial purposes [79]. Furthermore, monoacylglycerols seem to be overexpressed in salt stress conditions [80].

Concluding, all fatty acids have important roles in human physiology [81]. Halophytic grasses seem to be rich in these compounds, especially *A. donax*, since 21 fatty acids were reported in its extracts. This data supports the use of this species for alimentation purposes as well as a new source of bioactive molecules for the pharmaceutical industry. The requirement of fatty acids for resistance to salt stress could enhance the production of these compounds, making halophytic grasses a remarkable source of them. Nonetheless, more studies need to be conducted in order to fully comprehend the presence of this class of compounds in halophytes.

### 2.3. Cinnamic Acids, Benzoic Acids, and Other Short Chain Carboxylic Acids

**Figure 3:**
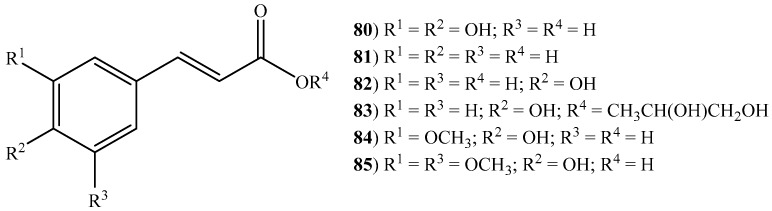
Some cinnamic acids and derivatives identified in halophytic grasses (see Table S1).

According to the literature, six cinnamic acids (**80**–**85**) were described among seven halophytic grasses (Figure 3): *B. dactyloides*, *C. dactylon*, *E. crus-galli*, *I. cylindrica*, *L. multiflorum*, *S. viridis*, and *Z. aquatica*. Regarding *C. dactylon* and *L. multiflorum*, four cinnamic acids were described [caffeic acid (**80**), cinnamic acid (**81**), *p*-coumaric acid (**82**) and ferulic acid (**84**)] (Table S1). In the first species, these were retrieved from the 80% aqueous ethanol and methanol extracts, [58,64,82,83], while in the second from the cell walls [84,85]. In the case of *I. cylindrica* [86,87,88], caffeic acid (**80**), *p*-coumaric acid (**82**), 1-*O*-*p*-coumaroylglycerol (**83**) and ferulic acid (**84**) were isolated from 70% aqueous ethanol extract. *p*-Coumaric acid (**82**) and ferulic acid (**84**) were also reported in the water extract of *B. dactyloides* [89] and isolated from the 70% aqueous ethanol extract of *E. crus-galli* [90] while in *S. viridis* only *p*-coumaric acid (**82**) was reported in the ethanol extract (Table S1) [91]. All the above mentioned cinnamic acids, with exception to 1-*O*-*p*-coumaroylglycerol were reported in the aqueous methanol extracts of *Z. aquatica* [55,92] (Table S1). Additionally, sinapic acid (**85**), known for its numerous biological activities including the ability to inhibit lipid peroxidation (IC_50_ (half minimum inhibitory concentration): 500μmol/kg) [93] and to promote anxiolytic effects (IC_50_: 4 mg/kg) [94], was also described in this species. Similarly, ferulic acid (**84**) and its derivatives are well known for their anti-inflammatory (IC_50_: 500 µg/mL) [95], antidiabetic (maximum concentration tested, 50 mg/(kg body weight·day)–1) [96], anti-carcinogenic (IC_50_: 25–75 µm) [97] anti-aging [98], and radioprotective (10 μM and 100 µM, dose-dependent) [99] properties, among others. These results along with the literature data about the medicinal attributes of cinnamic acids and derivatives [100] contribute to the halophytic grasses’ great potential to be used in food and pharmaceutical industries.

**Figure 4:**
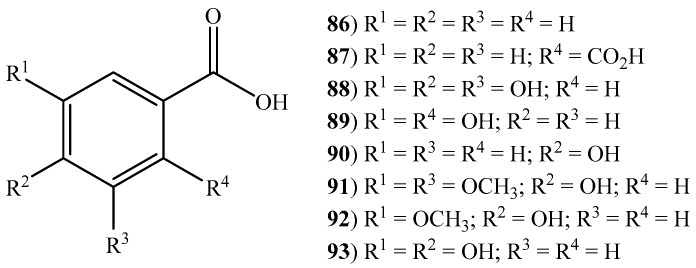
Some benzoic acids and derivatives identified in halophytic grasses (see Table S1).

Plant benzoic acids are considered to be building blocks or key structural elements for primary and specialized metabolites [101]. In halophytic grasses, nine benzoic acids and derivatives (**86**–**94**) (Figure 4) have been reported (Table S1). The species with greater variety were *E. crus-galli* and *Z. aquatica* with five compounds each. Protocatechuic acid (**94**), only reported in *Z. aquatica*, has been widely studied for its biological activities. A full review on this aspect was performed by Kakkar and co-workers (2014), emphasizing its antibacterial, antioxidant, antidiabetic, anticarcinogenic, antiviral, antiaging, antifibrotic, anti-inflammatory, antipyretic and analgesic effects [102].

Other short chain carboxylic acids and derivatives (**95**–**101**) were also reported in *C. dactylon*, *D. spicata*, *I. cylindrica*, *S. spontaneum*, and *Z. aquatica* (Table S1). These compounds are known for their wide range of pharmacological activities. For instance, citric acid (**96**), only reported in *C. dactylon*, have protective effects on myocardial ischemia/reperfusion injury (IC_50_: 400 μg/mL and 200 μg/mL) [103], while succinic acid (**101**) is known for its broad range of applications in the medicinal area as an antioxidant, antiradical and adaptogenic agent [104].

Cinnamic acids and derivatives, as well as short chain carboxylic acids, are common in diets rich in vegetables and fruits [105]. Its health benefits as antioxidants and diabetes-preventing molecules have been widely studied, as discussed above. Furthermore, some of the species with a vast array of cinnamic acids and derivatives reported, namely *E. crus-galli*, *I. cylindrica*, and *Z. aquatica*, are already considered edible [51,53,55]. Such status, allied with the advantages of a diet rich in this class of compounds highlights the benefits of their introduction in the human diet.

### 2.4. Carbohydrates and Amino Acids

Carbohydrates, 13 in total (**102**–**114**), were reported to be present in eight halophytes from the Poaceae: *A. donax*, *C. dactylon*, *D. spicata*, *I. cylindrica*, *P. maritima*, *S. spontaneum*, *S. anglica* and *S. pyramidalis* (Table S1). These compounds belong to several subclasses such as aldoses, ketoses, disaccharides, trisaccharides, tetrasaccharides, polysaccharides, and aldonic acids. *A. donax* has the most diverse carbohydrates reported (11 compounds), being the richest species regarding this class of compounds [43,106] (Table S1). *I. cylindrica* [107] and *S. pyramidalis* [108] contain 6 carbohydrates (Table S1), while in both *P. maritima* and *S. anglica* fructose, glucose and sucrose were reported [109]. Furthermore, in both *C. dactylon* [64] and *S. spontaneum* [59], only mannose was reported. At last, in *D. spicata*, sucrose was the only carbohydrate described [110]. These compounds were retrieved, in all cases from hydroalcoholic extracts. Sugars constitute a class of compounds which are energetic sources and add flavor to plants. Additionally, several health benefits are also being attributed to vegetal-derived sugars such as trehalose [111]. The presence of these molecules adds value to halophytic grasses and reveals their potential as functional foods.

Regarding amino acids (**115**–**131**), 17 were reported in three halophytic grasses, specifically in *D. bipinnata*, *S. anglica* and *P. maritima.* All 17 amino acids were reported in *D. bipinnata* perchloric acid (5% (*v*/*v*)) extract [110], while only two were described in *S. anglica* and *P. maritima*’s hydroalcoholic extracts [109] (Table S1). Amino acids are important compounds from a nutritional point of view since they are crucial to a balanced diet (maintaining optimal levels of essential amino acids) [112]. Some of the essential ones were reported in *D. bipinnata*, explicitly histidine, leucine, lysine, methionine, phenylalanine, threonine, and valine [110]. The remaining amino acids play an important role in plant physiology by maintaining homeostasis during osmotic stress due to the high concentration of NaCl in the external medium [5].

These two classes of compounds are indispensable from a nutritional point of view. The high diversity of amino acids in *D. bipinnata* could lead to its use as an alternative to other vegetal sources. Carbohydrates were reported to be well distributed among the studied halophytic grasses, which can also be a sign to their possible use as edible plants. Furthermore, due to its high diversity of carbohydrates (allied with their rich chemical composition), the introduction of *A. donax* in the diet should be considered after toxicological and safety assessments.

### 2.5. Terpenoids

Terpenoids are the class with more structural diversity among halophytic grasses summing 48 compounds distributed in several subclasses: triterpenoids (**167**–**180**), sesquiterpenoids (**181**–**189**), steroids and derivatives (**190**–**209**), diterpenoids (**216**), monoterpenoids (**211**–**215**) and tetraterpenoids (**210**) (Table S1). 

Triterpenoids were described in four halophytic grasses namely in *A. donax* (from lipophilic extracts), *C. ciliaris* (methanol, ethyl acetate, and hexane extracts), *C. dactylon* (80% aqueous ethanol extract) and *I. cylindrica* (chloroform/methanol extract) (Table S1). This subclass is well known for its pharmacological activities [113], for instance, α-amyrenone (**168**) and β-amyrenone (**167**), only reported in *A. donax*, have the ability to interfere in acute and chronic inflammatory processes (oral administration, 23.5 μmol/kg) [114]. Ursolic acid (**180**), found only in *A. donax*, possesses the ability to increase muscle mass and brown fat, leading to increased energy expenditure and therefore reduced obesity, improved glucose tolerance and decreased hepatic steatosis (high-fat diet supplemented with 0.14% ursolic acid, in rats) [115]. Lupeol (**177**), found only in *C. ciliaris*, revealed topic anti-inflammatory activity in mouse’s ears (topical administration, 0.5 and 1 mg/ear dose) [116] and anticarcinogenic activity against T-lymphoblastic leukaemia CEM (IC_50_: 50 µM), breast carcinoma MCF-7 (IC_50_: 50 µM), lung carcinoma A-549 (IC_50_: 50 µM), multiple myeloma RPMI 8226 (IC_50_: 50 µM), cervical carcinoma HeLa (IC_50_: 37 µM) and malignant melanoma G361 (IC_50_: 50 µM) when treated for 72 h [117]. The mentioned biological capacities of lupeol were performed in vivo and in vitro [118] fact that, in our opinion, increases its significance.

**Figure 5:**
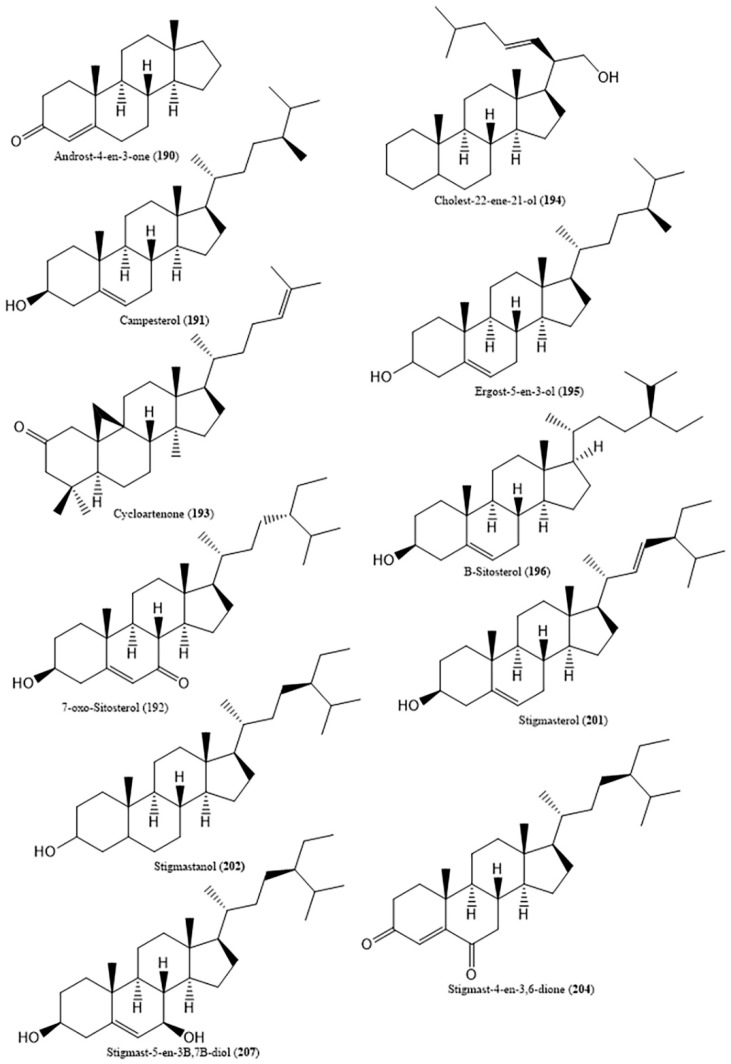
Some steroids and derivatives identified in halophytic grasses.

Among terpenoids found in halophytes from the Poaceae, steroids and their derivatives (**190**–**209**) (Figure 5) are the most reported ones. These were described in *A. donax*’s lipophilic extract (15 compounds), *C. ciliaris*’s methanol, ethyl acetate and hexane extracts (7 compounds), *C. dactylon*’s 80% aqueous ethanol extract (1 compounds), *D. bipinnata* (4 compounds) and *I. cylindrica*’s chloroform/methanol extracts (2 compounds). A diet rich in phytosterols is associated with lower risks of osteoporosis, heart disease, breast cancer, among others [119]. For instance, β-sitosterol (**196**) (reported in *A. donax*, *C. dactylon*, *D. bipinnata* and *I. cylindrica*) is known for its hypocholesterolemic activity [120] while their derivative β-sitosterol glucoside (**197**) (described in *A. donax*, *D. bipinnata*, and *I. cylindrica*) have antibacterial activity against *Escherichia coli* O157:H7 (EHEC) biofilms (IC_50_: 8.3 µM) [121]. Stigmasterol (**201**), present in *A. donax*, *C. ciliaris* and *D. bipinnata* proved its value as an antiasthmatic agent, with suppressive effects on essential features of allergen-induced asthma (dietary administered in guinea pigs, 10, 50, 100 mg/kg) [122].This compound also has the ability to protect pancreatic β-cells from glucotoxicity during diabetes progression through inhibition of early apoptosis, increasing total insulin and promoting insulin secretion [123].

Concerning monoterpenoids, 5 compounds (**211**–**215**) were reported in *C. ciliaris*, *C. dactylon*, *D. bipinnata* and *P. clandestinum*. This subclass is also known for the biological activities of its members [124]. For instance, α-pinene (**215**), only reported in *P. clandestinum*, show anti-inflammatory effects on human chondrocytes which leads to antiosteoarthritic activity [125]. This compound also displays antibacterial, antifungal and antibiofilm activities, with minimum inhibitory concentration (MIC) values ranging from 117 μg/mL to 4,150 μg/mL [126]. In the case of diterpenoids, only phytol (**210**) was reported in *C. ciliaris*, *C. dactylon*, and *S. spontaneum*. Nonetheless, phytol is well known for its apoptotic effects in human gastric cancer cells [127] as well as for its antioxidant activity [128]. Regarding sesquiterpenoids (**181**–**189**), these were reported in *C. dactylon*, *D. bipinnata*, *I. cylindrica*, *P. clandestinum*, and *S. spontaneum*.

Terpenoids was the most studied class among halophytic grasses. The compounds reported are widely diverse among the studied species and are known for their pharmacological activities. Moreover, the inclusion of food with a high quantity of these compounds is associated with chronic diseases’ prevention, highlighting the nutraceutical potential of these species.

### 2.6. Flavonoids

**Figure 6:**
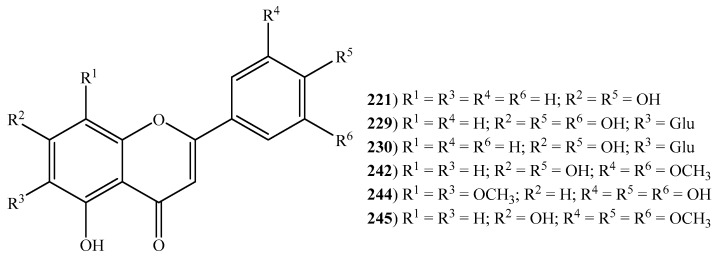
Some flavones identified in halophytic grasses (Glu = D-glucose) (see Table S1).

**Figure 7:**
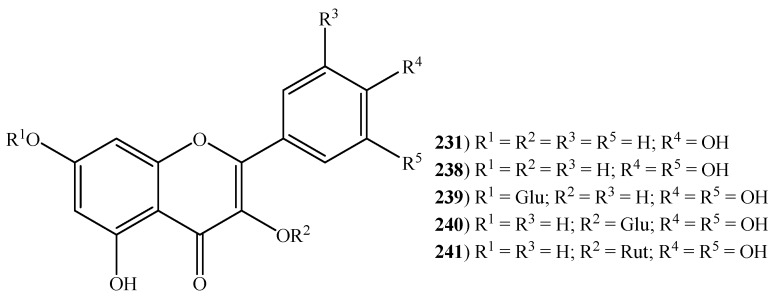
Some flavonols and derivatives reported in halophytic grasses (Glu = D-glucose; Rut = rutinose) (see Table S1).

Overall, 29 flavonoids (**211**–**249**) (Figure 6 and Figure 7) were reported in halophytic grasses, specifically 17 in *C. dactylon*’s aqueous and ethyl acetate extracts, seven in *Z. aquatica*’s phenolic fraction, three in *C. gayana*’s hexane extract, six in *D. bipinnata*’s ethanol extract, five in *E. crus-galli*’s 70% aqueous ethanol extract, four in *S. viridis*’s ethanol extract, three in *S. spontaneum*’s ethanol extract, two in *P. virgatum*’s aqueous extract and one isolated from roots of *I. cylindrica* (Table S1). Flavonoids are well-known for their pharmacological properties and their consumption is associated with reduced risk of several chronic illnesses such as cancer, cardiovascular diseases and neurodegenerative disorders [129]. For instance, the flavone apigenin (**221**), only reported in *C. dactylon*, is recognized for its anti-inflammatory (inhibition of E-selectin expression, IC_50_ 17.7 µM) [130], antianxiety (dietary administered, 10 mg/kg dose) [131] and anticarcinogenic activities [132,133]. The flavonoid epicatechin (**226**) (only described in *Z. aquatica*) proved their value as an anti-fatigue agent, by inducing structural and metabolic changes in skeletal and cardiac muscles, ultimately leading to endurance capacity (dietary administered, 1 mg/kg dose) [134]. This compound also possesses the ability to stimulate myocardial angiogenesis through rising protein levels and activation of canonical angiogenesis pathway (dietary administered, 1 mg/kg dose) [135]. Kaempferol (**231**), present in *C. dactylon*, *C. gayana*, *D. bipinnata*, and *Z. aquatica*, is well-known for its antimicrobial activity with MIC values ranging from 32 μg/mL to 512 μg/mL and antioxidant activity with an IC_50_ value of 52.48 μg/mL [136]. The anti-inflammatory property of kaempferol is linked to activity towards NF-κB (nuclear factor kappa-light-chain-enhancer of activated B cells) pathway proteins (IC_50_: 10 µM) [137]. This compound has also been able to inhibit the growth of HT-29 human colon cancer cells at a concentration of 60 µmol/L (IC_50_) [138]. The flavanone-7-*O*-glycoside, naringin (**235**), is reported to display antioxidant (IC_50_: 0.5 mg/mL) [139], anti-inflammatory (oral administered, 15.8 mg/kg/day dose) [140], antihypertensive (dietary administered, 20, 40 and 80 mg/kg doses) [141] and hypolipidemic activities [142]. The same activities are also recognized in quercetin (**238**) [143,144]. The flavonol glycoside, rutin (**241**) has a vast array of health benefits and pharmacological activities which includes antinociceptive, antarthritic, anti-diabetic, hypercholesteraemic, antiplatelet aggregation and antiasthmatic activities. A recent and complete review on this aspect, led by Ganeshpurkar and Saluja, (2017), is available [145]. These inherent properties of flavonoids categorize them as a class of beneficial compounds that have health-promoting and disease-preventing effects.

This is an important class of compounds from a pharmacological and nutritional point of view. There is evidence that points to lower risk of chronic diseases such as cancer and cardiovascular disorders for those who have a diet rich in flavonoids. Additionally, the species studied regarding this aspect showed a great diversity of compounds, enriching the nutraceutical value of halophytic grasses. 

### 2.7. Other Polyphenols

**Figure 8:**
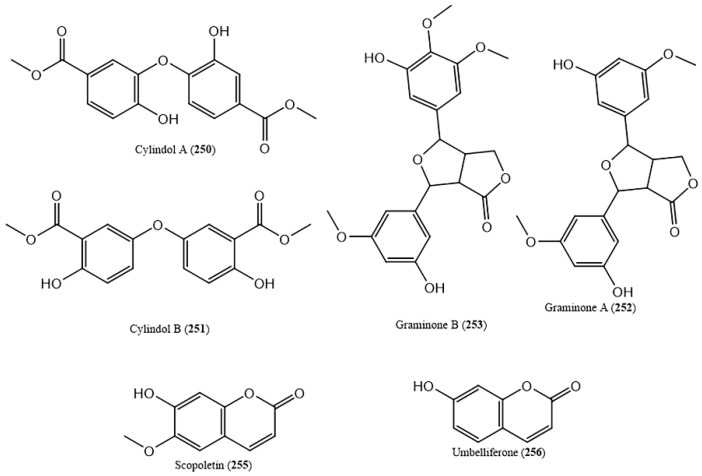
Some polyphenols reported in halophytic grasses.

Other polyphenols such as cylindol A (**250**) and B (**251**), imperanene (**254**) and graminone A (**252**) and B (**253**) (Table S1 and Figure 8) were also reported. In addition, coumarins (socopoletin, umbelliferone (Figure 8) and 4-methoxy-5-methylcoumarin-7-*O*-β-d-glucopyranoside) were isolated from *D. bipinnata* and *I. cylindrica* (Table S1). Socopoletin (**255**) has the ability to rescue impaired cholinergic functions (2 mg/kg sc, in rats) [146] while, umbelliferone (**256**), shows anticarcinogenic activities in colorectal cancer (intragastric injection at a daily dose of 30 mg/kg body weight) [147]. Additionally, it also shows great antinociceptive and anti-inflammatory activities due to the inhibition of peripherical and central acting pain mediators (5 and 10 mg/kg) [148].

Concluding, it is clear that these classes of compounds were not very explored in halophytic grasses with their presence only reported in 2% of the total diversity of the *taxa*. As the role of these compounds in the prevention of degenerative and cardiovascular diseases is evident, more studies on this aspect is recommendable [149].

### 2.8. Stilbenoids and Derivatives

According to the literature, 13 stilbenoids and derivatives (**272**–**284**) were described solely in the alcohol extract of C. dactylon [150]. These compounds are known for a wide spectrum of biological activities such as neuroprotection, cancer prevention, anti-obesity, anti-platelet aggregation, depigmentation, anti-diabetes and anti-atherosclerosis [151]. For instance, pallidol (**278**) has the ability to inhibit both cell growth in human cancer cells (HCT-116, HT29 and Caco-2 cell lines) [152] and the activity of protein kinase C [153], while parthenostilbenin A (**279**) and B (**280**) are able to inhibit lipid peroxidation (IC_50_ = 20.35 and 18.68, respectively) in rat liver homogenate [154]. Stilbenoids are normally found in tea, berries and wine at high concentrations [150] and their beneficial effects have been associated with antioxidant activity and thus, they have been considered of high nutraceutical value [155]. Therefore, the presence of these compounds in the extracts of C. dactylon is considerably interesting. 

### 2.9. Miscellaneous Compounds

In addition to the classes discussed above, other compounds belonging to miscellaneous chemical families were reported in halophytes from the Poaceae. Among these, two cyanogenic glucosides were described (Table S1). These compounds are toxic due to hydrogen cyanide release upon enzymatic breakdown and are present in more than 2650 plant species, including edible plants. Hydrogen cyanide derived from cyanogenic glucosides can lead to cell death through cytochrome oxidase blocking and ATP production arrestment. Symptoms of cyanogen poisoning include vomiting, nausea, dizziness, weakness, and occasionally death [156]. Two of these compounds were reported in *C. ciliaris* [58] (Table S1); nonetheless, no information is available concerning the quantities present in the plant and therefore, no assumption on edible safety can be made.

## 3. Biological Activities of Halophytic Grasses

Several halophytic grasses have been studied for their biological activities, with particular emphasis on their antibacterial, antifungal, antiviral, spasmolytic and antidiarrheal activities. Furthermore, anti-inflammatory, antioxidant, antidiabetic and anti-obesity properties, as well as anticarcinogenic and hepatoprotective effects, were also studied. In the case of *A. donax* and *C. dactylon*, extensive reviews about these aspects are already available. Al-Snafi compiled valuable information focusing on the different biological activities of *A.donax* [43]. Following the same path, Asthana and co-workers also revealed the potential of *C. dactylon* to be used in medicine [58]. Therefore, the pharmacological potentials of these species will not be discussed here. Besides these two species, 13 halophytic grasses, accounting 10% of the diversity of the *taxa*, were studied regarding their biological activities. Conversely, some of the species discussed in this chapter were not studied concerning their phytochemical composition, which is the case of *E. colona*, *Eleusine indica* (L.) Gaertn, *D. aegyptium*, *Phragmites australis* (Cav.) Trin. ex Steud. and *Phragmites karka* (Retz.) Trin. ex Steud.. The species with the greater number of described activities was *D. bipinnata* followed by *E. indica*, whose chemical composition is not known (Table S1). It is important to highlight that plant extracts should not be used without the knowledge of their chemical composition since the two aspects are inseparably linked because the pharmacological activities cannot be studied without knowledge of the substance present in the extracts. 

Some of the halophytic grasses with reports of their biological activities were already recognized for their use in traditional medicine. For instance, *D. bipinnata* has been used in Indian traditional medicine for the treatment of various disorders such as asthma, kidney stone, diarrhea and wound healing [157,158]. Similarly, *I. cylindrica* has been used for renal disorders [159] while *S. spontaneum* has been used for the treatment of mental illnesses as well as gastrointestinal disorders [160].

Even though an extensive description of the biological activities of these 13 species of halophytic grasses is available, in most cases, the compound(s) responsible for the medicinal properties as well as the mechanism of action are not known. A lack of information regarding the toxicologic effects of the extracts is evident, which is necessary for the correct use of medicinal plants. In this chapter, the reported biological activities of the extracts from halophytic grasses is described. 

### 3.1. Antibacterial, Antifungal And Antiviral Activities

Microorganisms are responsible for several important infectious diseases, and despite the progress in the development of antibacterial drugs it is still of great urgency to find new antibacterial agents capable of controlling multidrug resistance pathogens [161]. Several halophytic grasses are already known to possess antibacterial, antiviral and antifungal activities. For instance, polar extracts of *C. ciliaris* revealed significant antibacterial and antifungal activities against *Proteus merabilis* (MIC 0.234 mg/mL), *Klebsiella pnemoniae* (MIC 1.21 mg/mL) and *Agerobacterium tumefaciens* (MIC 4.24 mg/mL) [162]. Padalia and co-workers [163], through disk diffusion assay, evaluated different extracts of *S. spontaneum*’s aerial parts and concluded that the petroleum ether, ethyl acetate, acetone, and methanol extracts were active against gram-positive bacteria (inhibition zones of 10.0, 11.0, 12.5 and 10.0 mm, respectively) [163]. Some of the compounds found in *C. ciliaris* and *S. spontaneum* are well known for their antimicrobial activity; however, its non-polar nature [164] prevent their presence in polar extracts. This allowed us to conclude that only cholest-22-ene-21-ol could be present in the polar extract of *C. ciliaris* and thus, be responsible for its activity [56]. Therefore, more studies need to be conducted in order to identify the responsible compounds for the reported effects. 

The essential oils of *D. bipinnata* also exhibited antibacterial activity against *S. aureus*, *Staphylococcus epidermis*, *E. coli* and *P. aeruginosa* (MIC > 4 µg/mL) [160], while its ethanol extract inhibited *K. pneumonae* NCIM 2957 (MIC 0.977 mg/mL), *E. coli* NCIM 2931 (MIC 31.25 mg/mL), *Bacillus cereus* NCIM 2458 (MIC 31.25 mg/mL), *Salmonella typhimurium* NCIM 2501 (MIC 62.5 mg/mL) and *Proteus vulgaris* NCIM 2857 (MIC 62.5 mg/mL) [165]. In addition, the n-butanol extract of this species showed antibacterial activity against *Helicobacter pylori* with a MIC value of 6.25 mg/mL [166]. Kaempferol is reported to be present in the polar extracts of *D. bipinnata* [53] and their antimicrobial activity is widely recognized [167], which could be a clue for the compound(s) responsible for the activity of this extract. *E. crus-galli*’s methanol extract (1% acidified) also displayed antibacterial activity against *Bacillus megaterium* MTCC-428 (inhibition zone of 12 mm), *E. coli* MTCC 443 (inhibition zone of 16 mm) and *P. aeruginosa* MTCC1688 (inhibition zone of 13 mm). In contrast, its ethyl acetate extract inhibited *S. aureus* MTCC96 (inhibition zone of 14 mm) [168]. This activity could be related to *p*-coumaric acid and ferulic acid, both well-known antimicrobial agents [98,169] and present in the ethanol extract of *E. crus-galli* [90].

The methanol extracts of *P. karka* showed activity against *Actinobacter* sp. (inhibition zone of 9.4 mm), *Salmonella paratyphy* and *S. aureus* (inhibition zones of 10 mm), while its diethyl ether extract displayed activity against *E. coli* (9 mm) and *Klesebiella* sp. (9.7 mm) [170]. The ethyl acetate extract of *E. indica*, exhibited a wide spectrum of antibacterial activity against *S. aureus* (MRSA) (10 mm), *P. aeruginosa* 60690 (12 mm) and *Salmonella choleraesuis* (11 mm) [171], while its hexane extract showed a notable activity against *S. aureus* (13 mm) (MRSA) and *P. aeruginosa* (12 mm) [171]. The methanol extract of these species also presented protective effects against herpes simplex type 1 virus (HSV-1) infection, by inhibiting the docking of the virus in the surface of the cell as well as their penetration [172]. Regarding *E. colona*, its methanol and petroleum ether extracts were active against both gram-positive (*S. aureus* (21 mm) and *Streptococcus pneumoniae* (18 mm)) and gram-negative bacteria (*E. coli* (22 mm), and *P. aeruginosa* (19 mm)) as well as against fungal strains (*Aspergillus oryzae* (19 mm) and *A. niger* (48 mm)) [173]. The chemical composition of these three species is not known, and therefore no information is available regarding the potential active compounds.

The list of halophytic grasses with antibacterial activity is remarkable. Nonetheless, it is important to note that from 148 species of halophytic grasses only seven were evaluated for the discussed activities and that in all cases the active principles and mechanisms of action are not known.

### 3.2. Spasmolytic and Antidiarrheal Activities

Diseases related to the gastrointestinal system, specifically diarrhea and constipation, affect 70% of the population worldwide, with particular emphasis in developing countries [174]. Usually, medicinal plants are preferred to treat these disorders over synthetic formulations due to their multiple constituents, which can enhance action and/or neutralize side effects [175]. Three halophytic grasses were already studied regarding their spasmolytic and antidiarrheal activities.

*C. ciliaris* has been traditionally used to treat gastrointestinal disorders [176] and this practice has been confirmed by pharmacological studies proving their spasmolytic and antidiarrheal activities. Its ethanol extract showed dose-dependent protective effects against diarrhea and gastrointestinal motility (100 and 200 mg/kg) [45], which may be due to blockage of Ca^2+^ channels. In addition, this plant extract showed antiemetic activity (75, 100 and 150 mg/kg), which might be related to the presence of flavonoids, tannins, and alkaloids [45]. *D. bipinnata*’s ethanol extract displayed similar results; however, the active principles are still unknown [177]. Moreover, its hydroalcoholic extract, at doses of 200 mg/kg and 400 mg/kg, showed laxative and diuretic effects. The acute toxicity (LD_50_, median lethal dose) of *D. bipinnata*’s ethanol and hydroalcoholic extracts was assessed as 2000 mg/kg [177]. Several flavonoids known for their anti-inflammatory and antioxidant activities have been described in these species; nonetheless, the mechanisms of action, as well as the active principles, are still unknown [44].

Thespasmolytic effect (0.3–3.0 mg/mL) of the *D. aegyptium*’s ethanol extract was also hypothesized to be due to Ca^2+^ blocking components which ultimately causes relaxation of gastrointestinal smooth muscle, combating diarrhea [178]. Similarly to *C. ciliaris*, this species is also used traditionally to cope with these gastrointestinal disorders [178]. Once again, its chemical composition is still unknown.

### 3.3. Anti-Inflammatory and Antioxidant Effects

In recent years, the oxidative stress and its associated factors have gained importance in human health. This occurs due to the production of reactive oxygen species (ROS) (hydroxyl radicals, superoxide anion radicals and hydrogen peroxide) when the body is under stress [179]. This production results in imbalance processes, cell damage and health problems due to the overload of ROS that endogenous enzymatic and non-enzymatic antioxidant substances are not able to cope with [180]. This process often results in, among others, inflammatory diseases. Currently, inflammation is also one of the major researched areas for biomedical researchers [181]. The incorporation of antioxidant agents in diet from consumable natural plants can be used as preventive medicine for these disorders [181].

The ethanol and ethyl acetate extracts of *C. ciliaris* rhizome showed anti-inflammatory activities due to inhibition of cyclooxygenase-1 (COX-1) and cyclooxygenase-2 (COX-2) [176]. Compounds typical extracted by polar solvents, such as ethyl acetate, with anti-inflammatory activities were not reported in *C. ciliaris*, with the exception of cholest-22-ene-21-ol; hence, it is not possible to infer a secure relationship between the known chemical composition and the reported activity.

*D. bipinnata*’s ethanol extracts reduced significantly paw edema in rats (300 mg/kg), revealing its anti-inflammatory activity [182], while the roots’ methanol (70%) extract showed ROS scavenging activities assessed by H_2_O_2_ radical scavenging assay at concentrations of 50 μg/mL, 100 μg/mL, 200 μg/mL, 300 μg/mL, and 400 μg/mL [183]. These effects may be due to the presence of coumarin umbelliferone [53] and flavonol quercetin [53], which are present in the polar extracts of *D. bipinnata* and normally related to these activities [143,167]. Regarding antioxidant activity, this could also be related to quercetin as well as their glucoside derivatives (quercetin 3-*O*-glucoside and quercetin 7-*O*-glucoside), known for its antioxidant properties [184,185]. Similarly, the methanol and aqueous extracts of *E. crus-galli* showed strong antioxidant activity [168], assessed by DPPH (2,2-diphenyl-1-picrylhydrazyl) radical scavenging assay at concentrations of 50–500 μg/mL. This can also be attributed to quercetin and their 3-*O*-glucoside derivative but also to ferulic acid [186] and 5,7-dihydroxy-3′,4′,5′-trimethoxy flavone [187] known for their antioxidant properties. In the case of *C. gayana*, its isopropyl alcohol (3:2) extract showed free radical scavenging activity, concentration-dependent, in ABTS (2,2′-azino-bis(3-ethylbenzothiazoline-6-sulfonic acid) and DPPH assays and Fe^3+^ reducing potential until 500 µg/mL [68]. This can be related to the presence of the several flavonoids in its polar extracts [68], metabolites that are known for their antioxidant capacities [188,189,190].

*I. cylindrica*’s extracts are also known for their anti-inflammatory activities [191], which can be attributed to isoeugenin (Table S1), isolated from the methanol extract of this species’ roots. This compound was tested against macrophages (RAW264.7 cells) and showed significant activity (IC_50_ 9.33 µg/mL) in suppressing expressions at the mRNA (messenger ribonucleic acid) level of nitric oxide synthase (iNOS), cyclooxygenase-2 (COX-2) and proinflammatory cytokines [87]. Choi and co-workers (2017), conducted in vivo and in vitro assays to evaluate the anti-inflammatory activity of *L. multiflorum* methanol extract [192]. They concluded that this activity involves the suppression of NF-κB DNA-binding activation through inhibition of ERK (extracellular-signal-regulated kinase) and p38 MAPK (*mitogen-activated protein kinase*) phosphorylation. Although the polar components of this species are not well known, the activity could be related to ferulic acid [98], present in the polar extracts of *L. multiflorum* [84]

The ethanol root extract of *S. spontaneum* has the ability to scavenge free radicals (IC_50_ 488μg/mL) and therefore act as an antioxidant [193,194], which can also be due to the presence of quercetin [195]. *Z. aquatica* is claimed to hold antioxidant effects; therefore, Sumczynski and co-workers (2017) identified and evaluated the compounds that ultimately contribute to this property [92]. In their study, the activity was attributed to epigallocatechin, epicatechin, quercetin, and rutin as well as to ferulic acid, sinapic acid and other phenolic acids (Table S1) [92].

Through the data exposed above, it is possible to conclude that eight species of halophytic grasses were studied regarding their anti-inflammatory and antioxidant activities. Isoeugenin was the only compound directly linked to this effect even though the mechanisms of action were only grasped. Concerning antioxidant activity, only one study performed in *Z. aquatica* was able to enlighten the compounds that contribute the most to this effect. These investigations allow us to perceive the immense potential of halophytic grasses to treat and/or prevent inflammatory and oxidative-related disorders.

### 3.4. Anti-Diabetic and Anti-Obesity Activities

Recently, diabetes type 2 has been developing into a worldwide epidemic, mostly due to rapid economic growth and related lifestyle changes in the last 50 years [196]. This disorder is intimately related to obesity. It seems like the fast food culture allied with the sedentary lifestyle are the major causes of obesity, which contributes to insulin resistance and diabetes type 2 [197].

The methanol fraction of the ethanol extract from *D. aegyptium* (at a dose of 50 mg/kg) decreased both hyperglycemia and ameliorated oxidative stress, which contributed to its antidiabetic activities [198]. These could be related to insulinimimetic and antioxidant effects of the extract [198]. The mechanism of action, as well as the active principles, are still not known [198]. The methanol extract (70% methanol) of *D. bipinnata* was proven useful in the restoration of euglycemic levels (250 and 500 mg/kg) [157] while the hydroalcoholic extract of *E. crus-galli*’s grains exhibited significant antidiabetic activity in diabetic rats (400mg/kg and 200mg/kg) as well as antioxidant activity and both protection and regeneration of pancreatic β-cells [195]. Moreover, studies concerning acute toxicity showed that this extract does not cause major toxic effects [195]. These activities may be related to quercetin, which was reported to be present in *D. bipinnata* [53] and *E. crus-galli* [90] extracts and is recognized for its antidiabetic properties [144]. Likewise, the ethyl acetate root extract of *P. australis* was reported to have antidiabetic activity related to activation of peroxisome proliferator-activated receptor (PPARy). Nonetheless, the active principles and the exact mechanism of action are not known [199]. Ong and co-workers (2017) demonstrated the anti-obesity activity of *E. indica*’s methanol extract using obese rats; its properties seem to be due to pancreatic lipase inhibition (27.01 ± 5.68%) [200,201]. Similarly, the plant extract showed antidiabetic activities against diabetic rats [202]. 

Overall, the methanol extracts of halophytic grasses, as well as the hydroalcoholic and ethyl acetate extracts, were effective in the treatment of diabetes. However, the active principles and the mechanisms of action were not studied yet. *E. indica* was the only halophytic grass to be tested for its anti-obesity effects; nonetheless, the results seem promising.

### 3.5. Anticarcinogenic Activity

Cancer has become the second single cause of death, taking over six million lives every year around the world [203]. Recently, an urge to find new anticancer drugs from natural products has become evident [203] and investigations in plants have led to the discovery of many valuable compounds [203].

The methanol extract of *D. bipinnata* roots displayed dose-dependent anticarcinogenic activity (between 25–400 µg/mL) against human cervical cancer cell lines (HeLa), human laryngeal epithelial carcinoma cells (HEp-2), and mouse embryo fibroblast cells (NIH 3T3) [204]. The anticarcinogenic activity of the alkaloid umbelliferone [147] as well as of the flavonols kaempferol [205] and quercetin-3-*O*-glucoside [184] are well described. These compounds are present in *D. bipinnata*’s polar extract, which ultimately could contribute to the reported activity. *C. ciliaris* alcohol and successive extracts of both aerial and root parts were active against lung (A-549), intestinal (CACO), colon (HCT-116), cervical (Hela), hepatocellular (HepG-2) and breast (MCF-7) cancer cell lines with IC_50_ values between 11.1 ± 0.3 and 267 ± 0.8 µg/mL [206]. These activities might be related to the presence of lupeol and other sterols known for their anticarcinogenic activities and described on the polar extracts of *C. ciliatis* [117]

The ethanol extract of *E. crus-galli*’s seeds presented cytotoxic activity against four human cancer cell lines: colon (HCT-116) (IC_50_ = 11.2 ± 0.11µg/mL), cervical (HeLa) (IC_50_ = 12.0 ± 0.11 µg/mL), liver (HEPG-2) (IC_50_ = 14.2 ± 0.11 µg/mL) and breast (MCF-7) (IC_50_ = 18.9 ± 0.12 µg/mL) [90]. Methoxylated flavones present in this species’ extracts (Table S1) possess noteworthy cytotoxic activity, with IC_50_ values lower than the ones of the standard compound used [90]. Similarly, the butanol and hexane extract of *E. indica* inhibited the growth of human lung cancer (A549) and cervical cancer (HeLa) cells. Its hexane extract also induced apoptosis of A549 cells, indicating cytotoxic effects with IC_50_ values between 202 and 845 µg/mL, in both cases [207]. Similarly, the methanol extract of *I. cylindrica*’s leaves showed anticancer activity on oral cancer cell lines, inducing apoptosis of human tongue squamous cell carcinoma cells (SCC-9) [208]. This extract also showed cytotoxic activity against leukemia cell line CCRF-CEM in a dose-dependent manner [209]. These properties could be related to the cinnamic acids with anticarcinogenic activity [98,210] present in its extracts such as caffeic [86] and ferulic acids [87].

Overall, five halophytic grasses were tested concerning its anticancer properties against a large array of cancer cell lines and promising results were achieved in all of them. Nonetheless, more in vitro and in vivo studies are needed to fully characterize the active principles as well as to elucidate the mechanisms of action.

### 3.6. Hepatoprotective Activity

The liver is of extreme importance since it plays essential roles in the regulation of homeostasis and is frequently a target of numerous toxicants [211]. Although great advances have been made, in the field of hepatology, liver issues are still rising. In addition, only a few drugs with severe side effects are available for the treatment of liver disorders [211]. To cope with side effects, there is a growing interest in the study of medicinal plants, and halophytic grasses may be the answer to these issues.

*D. bipinnata* has proven to be hepatoprotective since its polyphenolic fraction was able to combat hepatoxicity in rats. This fraction is thought to protect the liver by free radical scavenging activity, ultimately leading to lipid peroxidation prevention at a dose of 100 mg/kg/day and 200 mg/kg/day [212]. Once again, the active principles are not known and the components of this polyphenolic fraction were not identified [212]. The methanol extract of *E. colona* also showed dose-dependent hepaprotective activity until 200 µg/mL in liver hepatocellular carcinoma (HepG2) cells [213]. Similarly, the aqueous extract of *E. indica* showed hepatoprotective effects against hepatoxicity in rats with IC_50_ of 2350 µg/mL [214]. Rehman and co-workers (2017) demonstrated the hepatoprotective activity of methanol extract of *I. cylindrical*, which was confirmed by the normalization of plasma markers after treatment with this extract [215]. Little information is known about the polar chemical composition of this species and therefore, no correlation between the phytoconstituents and the activity can be performed. 

Hepatoprotective activity has been observed with methanolic and aqueous extracts, as well as polyphenolic rich fractions of these halophytic grasses. In general, this activity appears to be linked with free radical scavenging abilities of the extracts. Nonetheless, more in vitro and in vitro studies are required to achieve a clear conclusion regarding the active principles and mechanisms of action.

### 3.7. Other Activities

The ethanol extract of *D. aegyptium* revealed antifertility activity, evident through reduced sperm count after treatment as well as reduced weight of reproductive organs and serum hormonal levels [216]. The compound(s) responsible for this activity are not known [216]. Additionally, the ethanol extracts of this plant exhibited antipyretic activities in rats (300 mg/kg) [182]. Daily oral treatment with aqueous extract of *D. bipinnata* (400 mg/kg) decreased the quantity of calcium oxalate deposited in the kidneys, resulting in anti-urolithiasis effects [217]. Ojha and co-workers (2010) evaluated the anticoagulant activity of *I cylindrica* methanol extracts (100, 200 and 400 mg/kg) and concluded that this may act on the extrinsic cascade of clotting by binding to antithrombin [218]. This extract increased prothrombin time significantly after first, second and third oral administration [218]. The methanol extract of *P. karka* also showed central nervous system depressant activity throught the reduction of sleep latency and increased duration of sleep [219]. The aqueous and ethanol extracts of *S. spontaneum* stems showed reduction in the motor activities of the rats tested, indicating central nervous system depressant properties [220]. The methanol extract from *I. cylindrica* roots exhibited dose-dependent anthelmintic activity (10–80 mg/50 mL) against *Pheretima nosthuma* (earthworms) compared with the control anthelmintic drug [221]. These extended lists of activities enlighten the great potential of halophytic grasses.

## 4. Conclusion, Discussion and Future Perspectives

In this review, we systematically went through the chemical composition and biological activities of halophytic grasses up to now. The identification of 300 compounds was summarized and present; among them, several chemical families were revealed (Table S1). Nonetheless, research on halophytes from Poaceae is still in its infancy and needs to be supplemented with further investigation on chemical composition, since only 14% of the diversity of the *taxa* was studied regarding this aspect. Moreover, the available information regarding some of the studied species is still scarce. This is evidenced by the different degree of compounds identified among the *taxa* so far, but also in the number of chemical classes described. For instance, in *B. dactyloides*, only cinnamic, benzoic and other short chain carboxylic acids and derivatives were described. Similarly, in *H. mucratom*, only fatty acids and derivatives were reported and in *S. pyramidalis* solely carbohydrates. *C. dactylon* is the only species with far more detailed characterization of its chemical composition (Figure 2) with 11 chemical families reported. In addition, from a chemical point of view, most of the studies lack a structural identification by nuclear magnetic resonance (NMR). Even with scarce studies, it is evident that halophytic grasses exhibit a wide range of chemical families, as well as a great diversity of compounds (Table S1), some of them being recognized for their biological activities with importance in pharmaceutical and nutritional areas. Nonetheless, only in-depth work on the phytochemical profile using modern chromatographic techniques such as high-performance liquid chromatography-mass spectrometry (HPLC-MS) and gas chromatography-mass spectrometry (GC-MS) as well as the isolation and characterization of the compounds will allow further speculation on their application in pharmaceutical and/or agrifood industry. Another important aspect considering the potential application of halophytic grasses in the food industry is the solvent used in the extraction process. For instance, carbon tetrachloride was used in the extraction of *S. spontaneum* and although in high yield, this solvent is considered highly carcinogenic leading to the formation of DNA adducts due to lipid peroxidation products formed during its metabolism [222]. Furthermore, it is also considered an environmental hazard since it contributes to the destruction of the ozone layer [223]. In food and pharmaceutical processing, only non-toxic solvents should be taken into consideration [224]; therefore, carbon tetrachloride has been banned in several countries, especially for use in consumable products [225]. It is also important to note that some of the reported compounds can be of fungal origin. Plant-associated microorganisms such as fungal endophytes are well described in grasses [226] and play important roles in the enhancement of salt stress resistance among halophytic species [227]. For instance, the genera *Epichloe* and *Neotyphodium* are well reported in the Poaceae [228] and several studies have associated the production of biological active compounds to these fungi [227] such as the case of ergot and lindole alkaloids and indole-diterpenes [229]. This is a point that needs to be taken into consideration while studying the chemical composition of halophytic grasses. For instance, *C. dactylon* present ergonovine (**262**) and ergonovinine (**263**) [58], two ergot alkaloids that might be produced by endophyte fungi association with this host.

Regarding the biological activities, the extracts and essential oils of halophytic grasses exhibit a wide spectrum of biological activities; nonetheless, the effects of the isolated compounds were not greatly explored in these species. In addition to this gap, the activity studies were only performed in 10% of the total diversity of halophytic grasses, which puts in evidence the lack of knowledge regarding these species. Furthermore, some halophytes from Poaceae with remarkable activities such as *E. colona*, *E. indica*, *D. aegyptium*, and *P. australis* have no information available regarding their chemical composition, which is essential to establish the modes of action. A link between the compounds reported in the *taxa* and their activity, when available, was made. Nonetheless, in some cases, few compounds were reported in the polar fraction, which was the most studied among all cases. Additionally, the activity of the extracts normally occurs due to the synergetic interaction of different compounds. At last, no study regarding the toxicity of the extracts as well as the mechanism of action responsible for the effect was performed. It is also important to highlight that, in some cases, the methods used to assess the activity of the extracts were not the best suited. For instance, in several cases, the method used to evaluate the antibacterial activity of the extracts was disk diffusion. Nonetheless, this presents many disadvantages: using volatile compounds could lead to reduced zones of inhibition and poorly soluble compounds do not diffuse uniformly through the agar matrix [230]. Moreover, for non-polar extracts and essential oils, diffusion techniques are not suitable at all since the compounds will not diffuse through the media. [230]. In these cases, the golden standard is considered to be the broth microdilution assay [231]. Therefore, there is an urge for uniformization of the methods applied to evaluate the biological activities of the extracts and essential oils.

Concluding, the findings in the reviewed papers highlighted the great potential of halophytes from Poaceae as sources of bioactive molecules as well as biological properties and an opportunity for development of value-added products for nutraceuticals and food applications. In addition to the health benefits that this species might bring, it also secures the future of modern agriculture due to increased soil salinity. Nonetheless, halophytic grasses are yet to be explored since these investigations were only conducted in few *taxa*. Furthermore, several gaps in our understanding of its application still exist. The biological properties revealed in the different extracts need to be complemented with research into clinical application. Although no serious marked effects have been reported in these species, further toxicity and safety evaluation of the extracts and chemical compounds isolated from the species should be carried out.

## Supplementary Materials

Supplementary materials can be found at https://www.mdpi.com/1422-0067/20/5/1067/s1. Reference [232,233,234,235,236,237,238,239,240,241,242,243,244,245] are cited in the supplementary materials.

## Abbreviations

ABTS	2,2’-Azino-bis(3-ethylbenzothiazoline-6-sulfonic acid
DPPH	2,2-Diphenyl-1-picrylhydrazyl
ERK	Extracellular-signal-regulated kinase
IC_50_	Half minimum inhibitory concentration
LD_50_	Median lethal dose
MIC	Minimum inhibitory concentration
MAPK	*Mitogen-activated protein kinase*
mRNA	Messenger ribonucleic acid
NF-kβ	Nuclear factor kappa-light-chain-enhancer of activated B cells
PPAR	Peroxisome proliferator-activated receptor
PUFA	Polyunsaturated fatty acids
ROS	Reactive oxygen species
