# Supplementary material for: Halophytic Grasses, a New Source of Nutraceuticals? A Review on Their Secondary Metabolites and Biological Activities
Source: Int J Mol Sci. 2019 Mar 1;20(5):1067. doi: 10.3390/ijms20051067 (PMC6429475; doi:10.3390/ijms20051067)
Supplement: Supplementary file 1 [file ijms-20-01067-s001.pdf]

Nr.		*Arundo donax*	*Buchloe dactyloides*	*Cenchrus ciliaris*	*Chloris gayana*	*Cynodon dactylon*	*Desmostachya bipinnata*	*Distichlis spicata*	*Echinochloa crus-galli*	*Halopyrum mucronatum*	*Imperata Cylindrica*	*Lolium multiflorum*	*Panicum virgatum*	*Pennisetum clandestinum*	*Puccinellia maritima*	*Saccharum spontaneum*	*Setaria viridis*	*Spartina anglica*	*Spartina patens*	*Sporobolus pyramidalis*	*Zizania aquatica*	*References*
	**Alkanes and Alkenes**																					
**1**	Propane (C_2)	nd	nd	nd	nd	nd	nd	nd	nd	nd	nd	nd	nd	nd	nd	+	nd	nd	nd	nd	nd	[59]
**2**	Hexane (C_6)	nd	nd	+	nd	nd	nd	nd	nd	nd	nd	nd	nd	nd	nd	nd	nd	nd	nd	nd	nd	[57]
**3**	Undecane (C_{11})	nd	nd	+	nd	nd	nd	nd	nd	nd	nd	nd	nd	nd	nd	nd	nd	nd	nd	nd	nd	[57]
**4**	Tridecane (C_{13})	nd	nd	+	nd	nd	nd	nd	nd	nd	nd	nd	nd	nd	nd	nd	nd	nd	nd	nd	nd	[56]
**5**	Tetradecane (C_{14})	nd	nd	+	nd	nd	nd	nd	nd	nd	nd	nd	nd	nd	nd	nd	nd	nd	nd	nd	nd	[56]
**6**	Pentadecane (C_{15})	nd	nd	+	nd	nd	nd	nd	nd	nd	nd	nd	nd	nd	nd	nd	nd	nd	nd	nd	nd	[56]
**7**	Hexadecane (C_{16})	nd	nd	+	nd	nd	nd	nd	nd	nd	nd	nd	nd	nd	nd	nd	nd	nd	nd	nd	nd	[56]
**8**	Heptadecane (C_{17})	nd	nd	+	nd	nd	nd	nd	nd	nd	nd	nd	nd	nd	nd	nd	nd	nd	nd	nd	nd	[56]
**9**	Octadecane (C_{18})	nd	nd	+	nd	nd	nd	nd	nd	nd	nd	nd	nd	nd	nd	nd	nd	nd	nd	nd	nd	[56]
**10**	Nonadecane (C_{19})	nd	nd	+	nd	nd	nd	nd	nd	nd	nd	nd	nd	nd	nd	nd	nd	nd	nd	nd	nd	[56]
**11**	Eicosane (C_{20})	nd	nd	+	nd	nd	nd	nd	nd	nd	nd	nd	nd	nd	nd	nd	nd	nd	nd	nd	nd	[56]
**12**	Heneicosane (C_{21})	nd	nd	+	nd	nd	nd	nd	nd	nd	nd	nd	nd	nd	nd	nd	nd	nd	nd	nd	nd	[56]
**13**	Docosane (C_{22})	+	nd	nd	nd	nd	nd	nd	nd	nd	nd	nd	nd	nd	nd	nd	nd	nd	nd	nd	nd	[43]
**14**	Tricosane (C_{23})	+[a]	nd	+[b]	nd	nd	nd	nd	nd	nd	nd	nd	nd	nd	nd	nd	nd	nd	nd	nd	nd	[a][43]; [b][56]
**15**	Tetracosane (C_{24})	+[a]	nd	+[b]	nd	nd	nd	nd	nd	nd	nd	nd	nd	nd	nd	nd	nd	nd	nd	nd	nd	[a][43]; [b][56]
**16**	Pentacosane (C_{25})	+	nd	nd	nd	nd	nd	nd	nd	nd	nd	nd	nd	nd	nd	nd	nd	nd	nd	nd	nd	[43]
**17**	Hexacosane (C_{26})	+	nd	nd	nd	nd	nd	nd	nd	nd	nd	nd	nd	nd	nd	nd	nd	nd	nd	nd	nd	[43]
**18**	Heptacosane (C_{27})	+	nd	nd	nd	nd	nd	nd	nd	nd	nd	nd	nd	nd	nd	nd	nd	nd	nd	nd	nd	[43]
**19**	Octacosane (C_{28})	+	nd	nd	nd	nd	nd	nd	nd	nd	nd	nd	nd	nd	nd	nd	nd	nd	nd	nd	nd	[43]

**20**	Nonacosane (C_{29})	+	nd	nd	nd	nd	nd	nd	nd	nd	nd	nd	nd	nd	nd	nd	nd	nd	nd	nd	nd	[43]
**21**	Triacontane (C_{30})	+	nd	nd	nd	+	nd	nd	nd	nd	nd	nd	nd	nd	nd	nd	nd	nd	nd	nd	nd	[a][43]; [b][58]
**22**	Hentriacontane (C_{31})	+	nd	nd	nd	nd	nd	nd	nd	nd	nd	nd	nd	nd	nd	nd	nd	nd	nd	nd	nd	[43]
**23**	Dotriacontane (C_{32})	+	nd	nd	nd	nd	nd	nd	nd	nd	nd	nd	nd	nd	nd	nd	nd	nd	nd	nd	nd	[43]
**24**	Tritriacontane (C_{33})	+	nd	nd	nd	nd	nd	nd	nd	nd	nd	nd	nd	nd	nd	nd	nd	nd	nd	nd	nd	[43]
**25**	Tetracontane (C_{34})	nd	nd	+	nd	nd	nd	nd	nd	nd	nd	nd	nd	nd	nd	nd	nd	nd	nd	nd	nd	[56]
**26**	Pentatriacontane (C_{35})	nd	nd	+	nd	nd	nd	nd	nd	nd	nd	nd	nd	nd	nd	nd	nd	nd	nd	nd	nd	[56]
**27**	Tetratetracontane (C_{44})	nd	nd	+	nd	nd	nd	nd	nd	nd	nd	nd	nd	nd	nd	nd	nd	nd	nd	nd	nd	[56]
**28**	Tetradec-1-ene	nd	nd	+	nd	nd	nd	nd	nd	nd	nd	nd	nd	nd	nd	nd	nd	nd	nd	nd	nd	[56]
**29**	2-Bromo-eicos-9-ene*	nd	nd	+	nd	nd	nd	nd	nd	nd	nd	nd	nd	nd	nd	nd	nd	nd	nd	nd	nd	[56]
	**Fatty acids, Acylglycerols and Derivatives**																					
**30**	Pentanoic acid (Valeric acid)	nd	nd	+[a]	nd	+[b]	nd	nd	nd	nd	nd	nd	nd	nd	nd	nd	nd	nd	nd	nd	nd	[a][57] [b][64]
**31**	Hexanoic acid (Caproic acid)	nd	nd	+	nd	nd	nd	nd	nd	nd	nd	nd	nd	nd	nd	nd	nd	nd	nd	nd	nd	[56]
**32**	Octanoic acid (Caprylic acid)	nd	nd	nd	nd	nd	nd	nd	nd	nd	nd	+[b]	nd	nd	nd	+[c]	nd	nd	nd	nd	nd	[a][68]; [b][232]; [c][59]
**33**	Non-6-enoic acid	nd	nd	nd	nd	nd	nd	nd	nd	nd	nd	nd	nd	nd	nd	+	nd	nd	nd	nd	nd	[59]
**34**	Decanoic acid (Capric acid)	nd	nd	nd	+[a]	+[b]	nd	nd	nd	nd	nd	nd	nd	nd	nd	nd	nd	nd	nd	nd	nd	[a][68]; [b][64]
**35**	Dodecanoic acid (Lauric acid)	+[a]	nd	+[b]	+[c]	nd	nd	nd	nd	nd	nd	nd	nd	nd	nd	nd	nd	nd	nd	nd	nd	[a][233]; [b][57]; [c][68]
**36**	Tetradecanoic acid (Myristic acid)	+[a]	nd	nd	+[b]	nd	nd	nd	nd	nd	nd	nd	nd	nd	nd	+[c]	nd	nd	nd	nd	nd	[a][43]; [b][68]; [c][59]
37	Ethyl pentadecanoic acid	nd	nd	nd	nd	+	nd	nd	nd	nd	nd	nd	nd	nd	nd	nd	nd	nd	nd	nd	nd	[65]
38	Pentadecanoic acid	+[a]	nd	+[b]	nd	nd	nd	nd	nd	+[c]	nd	nd	nd	nd	nd	Nd	nd	nd	nd	nd	nd	[a][43]; [b][56]; [c][67]
39	Pentadecenoic acid	nd	nd	+[a]	nd	nd	nd	nd	nd	nd	nd	nd	nd	nd	nd	+[b]	nd	nd	nd	nd	nd	[a][56]; [b][59]
40	Ethyl hexadecanoate	nd	nd	nd	+	nd	nd	nd	nd	nd	nd	nd	nd	nd	nd	nd	nd	nd	nd	nd	nd	[65]

41	Hexadecanoic acid (Palmitic acid)	+[a]	nd	+[b]	+[c]	+[d]	nd	nd	nd	+[e]	nd	nd	nd	nd	nd	nd	nd	nd	+[f]	nd	nd	[a][43];[b][56,57], [c][68]; [d][58,64–66]; [e][67]; [f][69]
42	Hexadec-9-enoic acid (Palmitoleic acid)	nd	nd	nd	nd	nd	nd	nd	nd	nd	nd	nd	nd	nd	nd	nd	nd	nd	+	nd	nd	[69]
43	Heptadecanoic acid (Margaric acid)	+[a]	nd	+[b]	nd	nd	nd	nd	nd	nd	nd	nd	nd	nd	nd	nd	nd	nd	nd	nd	nd	[a][43]; [b][56]
44	Heptadecenoic acid	nd	nd	nd	nd	nd	nd	nd	nd	nd	nd	nd	nd	nd	nd	+	nd	nd	nd	nd	nd	[59]
45	Ethyl octadecanoate	nd	nd	nd	nd	+	nd	nd	nd	nd	nd	nd	nd	nd	nd	nd	nd	nd	nd	nd	nd	[65]
46	Octadecanoic acid (Stearic acid)	+[a]	nd	+[b]	+[c]	+[d]	nd	nd	nd	+[e]	nd	nd	nd	nd	nd	+[f]	nd	nd	+[g]	nd	nd	[a][43]; [b][56]; [c][68]; [d][64]; [e][67]; [f][59]; [g][69]
47	Ethyl octadecenoate	nd	nd	nd	nd	+	nd	nd	nd	nd	nd	nd	nd	nd	nd	nd	nd	nd	nd	nd	nd	[65]
**48**	Octadecenoic acid (Oleic acid)	nd	nd	+[a]	+[b]	nd	nd	nd	nd	nd	nd	+[c]	nd	nd	nd	+[d]	nd	nd	nd	nd	nd	[a][56];[b][68]; [c][232];[d][59]
**49**	Ethyl Octadeca-9,12-dienate	nd	nd	nd	nd	+	nd	nd	nd	nd	nd	nd	nd	nd	nd	nd	nd	nd	nd	nd	nd	[65]
**50**	Octadeca-9,12-dienoic acid (Linoleic Acid)	+[a]	nd	nd	+[b]	+[c]	nd	nd	nd	nd	nd	nd	nd	nd	nd	+[d]	nd	nd	+[e]	nd	nd	[a][43]; [b][68]; [c][65];[d][59]; [e][69]
**51**	Octadeca-8,11-dienoic acid	nd	nd	nd	nd	+[a]	nd	nd	nd	+[b]	nd	nd	nd	nd	nd	nd	nd	nd	nd	nd	nd	[a][64]; [b][67]
**52**	Octadeca-9,12,15-trienoic acid (Linolenic acid)	nd	nd	nd	nd	+[a]	nd	nd	nd	nd	nd	+[b]	nd	nd	nd	+[c]	nd	nd	+[d]	nd	nd	[a][64]; [b][232]; [c][59]; [d][69]
**53**	Nonadecanoic acid	+	nd	nd	nd	nd	nd	nd	nd	nd	nd	nd	nd	nd	nd	nd	nd	nd	nd	nd	nd	[43]
**54**	Eicosanoic acid (Arachidic acid)	+[a]	-	+[b]	nd	+[c]	-	-	-	+[d]	nd	nd	nd	nd	nd	nd	nd	nd	nd	nd	nd	[a][43]; [b][56]; [c][58]; [d][67]
**55**	Heneicosanoic acid	+	nd	nd	nd	nd	nd	nd	nd	nd	nd	nd	nd	nd	nd	nd	nd	nd	nd	nd	nd	[43]
56	Docosanoic acid (Behenic acid)	+[a]	nd	nd	nd	+[b]	nd	nd	nd	+[c]	nd	nd	nd	nd	nd	+[d]	nd	nd	nd	nd	nd	[a][43]; [b][58]; [c][67]; [d][59]

**57**	Docosahexaenoic acid (Cervonic acid)	nd	nd	nd	nd	nd	nd	nd	nd	nd	nd	+	nd	nd	nd	nd	nd	nd	nd	nd	nd	[232]
**58**	Docosatetraenoic acid (Adrenic acid)	nd	nd	nd	nd	nd	nd	nd	nd	nd	nd	+	nd	nd	nd	nd	nd	nd	nd	nd	nd	[232]
**59**	Tricosanoic acid	+	nd	nd	nd	nd	nd	nd	nd	nd	nd	nd	nd	nd	nd	nd	nd	nd	nd	nd	nd	[43]
**60**	Tetracosanoic acid (Lignoceric acid)	+[a]	nd	nd	nd	nd	nd	nd	nd	+[b]	nd	nd	nd	nd	nd	nd	nd	nd	nd	nd	nd	[a][43]; [b][67]
**61**	Pentacosanoic acid (Hyenic acid)	+	nd	nd	nd	nd	nd	nd	nd	nd	nd	nd	nd	nd	nd	nd	nd	nd	nd	nd	nd	[43]
**62**	Hexacosanoic acid (Cerotic acid)	+[a]	nd	nd	nd	nd	nd	nd	nd	+[b]	nd	nd	nd	nd	nd	nd	nd	nd	nd	nd	nd	[a][43]; [b][67]
**63**	Heptacosanoic acid (Carboceric acid)	+	nd	nd	nd	nd	nd	nd	nd	nd	nd	nd	nd	nd	nd	nd	nd	nd	nd	nd	nd	[43]
**64**	Octacosanoic acid (Montanic acid)	+	nd	nd	nd	nd	nd	nd	nd	nd	nd	nd	nd	nd	nd	nd	nd	nd	nd	nd	nd	[43]
**65**	Nonacosanoic acid	+	nd	nd	nd	nd	nd	nd	nd	nd	nd	nd	nd	nd	nd	nd	nd	nd	nd	nd	nd	[43]
**66**	Triacontanoic acid (Melissic acid)	+	nd	nd	nd	nd	nd	nd	nd	nd	nd	nd	nd	nd	nd	nd	nd	nd	nd	nd	nd	[43]
**67**	Hentriacontanoic acid	+	nd	nd	nd	nd	nd	nd	nd	nd	nd	nd	nd	nd	nd	nd	nd	nd	nd	nd	nd	[43]
**68**	Dotriacontanoic acid (Lacceroic acid)	+	nd	nd	nd	nd	nd	nd	nd	nd	nd	nd	nd	nd	nd	nd	nd	nd	nd	nd	nd	[43]
**69**	2,3-Dihydroxypropyl tetradecanoate	+	nd	nd	nd	nd	nd	nd	nd	nd	nd	nd	nd	nd	nd	nd	nd	nd	nd	nd	nd	[43]
**70**	2,3-Dihydroxypropyl hexadecanoate	+	nd	nd	nd	nd	nd	nd	nd	nd	nd	nd	nd	nd	nd	nd	nd	nd	nd	nd	nd	[43]
**71**	2,3-Dihydroxypropyl octadecanoate	+	nd	nd	nd	nd	nd	nd	nd	nd	nd	nd	nd	nd	nd	nd	nd	nd	nd	nd	nd	[43]
**72**	2,3-Dihydroxypropyl eicosanoate	+	nd	nd	nd	nd	nd	nd	nd	nd	nd	nd	nd	nd	nd	nd	nd	nd	nd	nd	nd	[43]
**73**	2,3-Dihydroxypropyl docosanoate	+	nd	nd	nd	nd	nd	nd	nd	nd	nd	nd	nd	nd	nd	nd	nd	nd	nd	nd	nd	[43]

**74**	2,3-Dihydroxypropyl tetracosanoate	+	nd	nd	nd	nd	nd	nd	nd	nd	nd	nd	nd	nd	nd	nd	nd	nd	nd	nd	nd	[43]
**75**	2,3-Dihydroxypropyl hexacosanoate	+	nd	nd	nd	nd	nd	nd	nd	nd	nd	nd	nd	nd	nd	nd	nd	nd	nd	nd	nd	[43]
**76**	Dipalmitin	+	nd	nd	nd	nd	nd	nd	nd	nd	nd	nd	nd	nd	nd	nd	nd	nd	nd	nd	nd	[43]
**77**	Dipalmitin, 1,3-(P2)	+	nd	nd	nd	nd	nd	nd	nd	nd	nd	nd	nd	nd	nd	nd	nd	nd	nd	nd	nd	[43]
**78**	Distearin	+	nd	nd	nd	nd	nd	nd	nd	nd	nd	nd	nd	nd	nd	nd	nd	nd	nd	nd	nd	[43]
**79**	Palmitoylstearin	+	nd	nd	nd	nd	nd	nd	nd	nd	nd	nd	nd	nd	nd	nd	nd	nd	nd	nd	nd	[43]
	**Cinnamic acids and derivatives**																					
**80**	Caffeic acid	nd	nd	nd	nd	+[a]	nd	nd	nd	nd	+[b]	+[c]	nd	nd	nd	nd	nd	nd	nd	nd	+[d]	[a][82]; [b][86]; [c][85]; [d][92]
**81**	Cinnamic acid	nd	nd	nd	nd	+[a]	nd	nd	nd	nd	nd	+[b]	nd	nd	nd	nd	nd	nd	nd	nd	+[c]	[a][64,83]; [b][85]; [c][92]
**82**	*p*-Coumaric acid	nd	+[a]	nd	nd	+[b]	nd	nd	+[c]	nd	+[d]	+[e]	nd	nd	nd	nd	+[f]	nd	nd	nd	+[g]	[a][89]; [b][58,82,83]; [c][90]; [d][87]; [e][84,85]; [f][91]; [g][55,92]
**83**	1-*O*-*p*-coumaroylglycerol	nd	nd	nd	nd	nd	nd	nd	nd	nd	+	nd	nd	nd	nd	nd	nd	nd	nd	nd	nd	[88]
**84**	Ferulic acid	nd	+[a]	nd	nd	+[b]	nd	nd	+[c]	nd	+[d]	+[e]	nd	nd	nd	nd	nd	nd	nd	nd	+[f]	[a][89]; [b][58,234]; [c][90]; [d][87]; [e][84]; [f][55,92]
**85**	Sinapic acid	nd	nd	nd	nd	nd	nd	nd	nd	nd	nd	nd	nd	nd	nd	nd	nd	nd	nd	nd	+	[55,92]
	**Benzoic acids and Derivatives**																					
**86**	Benzoic acid	nd	nd	nd	nd	+	nd	nd	nd	nd	nd	nd	nd	nd	nd	nd	nd	nd	nd	nd	nd	[64]
**87**	1,2-Benzenedicarboxylic acid	nd	nd	+[a]	nd	nd	nd	nd	nd	nd	nd	nd	nd	nd	nd	+[b]	nd	nd	nd	nd	nd	[a][56]; [b][59]
**88**	Gallic acid	nd	nd	nd	nd	+[a]	nd	nd	nd	nd	nd	nd	nd	nd	nd	nd	nd	nd	nd	nd	+[b]	[a][82]; [b][92]
**89**	Gentisic acid	nd	+	nd	nd	nd	nd	nd	nd	nd	nd	nd	nd	nd	nd	nd	nd	nd	nd	nd	nd	[89]
**90**	*p*-Hydroxybenzoic acid	nd	+[a]	nd	nd	+[b]	nd	nd	nd	nd	+[c]	+[d]	nd	nd	nd	nd	nd	nd	nd	nd	+[e]	[a][89];

																						[b][58,82,234]; [c][88]; [d][84]; [e][92]
**91**	Homoveratric acid	nd	+	nd	nd	nd	nd	nd	nd	nd	nd	nd	nd	nd	nd	nd	nd	nd	nd	nd	nd	[89]
**92**	Syringic acid	nd	nd	nd	nd	+[a]	nd	nd	nd	nd	nd	+[b]	nd	nd	nd	nd	nd	nd	nd	nd	+[c]	[a][58,64,82]; [b][84]; [c][92]
**93**	Vanillic acid	nd	+[a]	nd	nd	+[b]	nd	nd	nd	nd	+[c]	+[d]	nd	nd	nd	nd	nd	nd	nd	nd	+[e]	[a][89];[b][58,64,234]; [c][88];[d][84]; [e][92]
**94**	Protocatechuic acid	nd	nd	nd	nd	nd	nd	nd	nd	nd	nd	nd	nd	nd	nd	nd	nd	nd	nd	nd	+	[92]
	**Other Short Chain Carboxylic Acids and Derivatives**																					
**95**	Ascorbic acid	nd	nd	nd	nd	+[a]	nd	+[b]	nd	nd	nd	nd	nd	nd	nd	nd	nd	nd	nd	nd	nd	[a][82]; [b][110]
**96**	Citric acid	nd	nd	nd	nd	nd	nd	+	nd	nd	nd	nd	nd	nd	nd	nd	nd	nd	nd	nd	nd	[110]
	Chlorogenic acid	nd	nd	nd	nd	nd	nd	nd	nd	nd	nd	nd	nd	nd	nd	nd	nd	nd	nd	nd	+	[92]
**97**	Dihydroxybutyric acid	nd	nd	nd	nd	nd	nd	nd	nd	nd	+	nd	nd	nd	nd	nd	nd	nd	nd	nd	nd	[88]
**98**	Lactic acid	nd	nd	nd	nd	nd	nd	+	nd	nd	nd	nd	nd	nd	nd	nd	nd	nd	nd	nd	nd	[110]
**99**	Propanoic acid	nd	nd	nd	nd	+[a]	nd	nd	nd	nd	nd	nd	nd	nd	nd	+[b]	nd	nd	nd	nd	nd	[a][64]; [b][59]
**100**	Propanedioic acid (Malonic acid)	nd	nd	nd	nd	+	nd	nd	nd	nd	nd	nd	nd	nd	nd	nd	nd	nd	nd	nd	nd	[64]
**101**	Succinic acid	nd	nd	nd	nd	nd	nd	+	nd	nd	nd	nd	nd	nd	nd	nd	nd	nd	nd	nd	nd	[110]
	**Carbohydrates**																					
**102**	Arabinose	+[a]	nd	nd	nd	nd	nd	nd	nd	nd	+[b]	nd	nd	nd	nd	nd	nd	nd	nd	nd	nd	[a][43]; [b][107]
**103**	Xylose	+[a]	nd	nd	nd	nd	nd	nd	nd	nd	+[b]	nd	nd	nd	nd	nd	nd	nd	nd	nd	nd	[a][235]; [b][107]
**104**	Fructose	+[a]	nd	nd	nd	nd	nd	nd	nd	nd	nd	nd	nd	nd	+[b]	nd	nd	+[c]	nd	+[d]	nd	[a][106]; [b, c] [109]; [d][108]
**105**	Galactose	+[a]	nd	nd	nd	nd	nd	nd	nd	nd	+[b]	nd	nd	nd	nd	nd	nd	nd	nd	nd	nd	[a][106]; [b][107]
**106**	Glucose	+[a]	nd	nd	nd	nd	nd	nd	nd	nd	+[b]	nd	nd	nd	+[c]	nd	nd	+[d]	nd	+[e]	nd	[a][106]; [b][107]; [c, d] [109]; [e][108]
**107**	Mannose	+[a]	nd	nd	nd	+[b]	nd	nd	nd	nd	+[c]	nd	nd	nd	nd	+[d]	nd	nd	nd	nd	nd	[a][43]; [b][64]; [c][107]; [d][59]
**108**	Rhamnose	+[a]	nd	nd	nd	nd	nd	nd	nd	nd	+[b]	nd	nd	nd	nd	nd	nd	nd	nd	nd	nd	[a][43]; [b][107]
**109**	Sucrose	+[a]	nd	nd	nd	nd	nd	+[b]	nd	nd	nd	nd	nd	nd	+[c]	nd	nd	+[d]	nd	+[e]	nd	[a][106]; [b][110]

																						[c, d][109]; [e][108]
**110**	Trehalose	-	nd	nd	nd	nd	nd	nd	nd	nd	nd	nd	nd	nd	nd	nd	nd	nd	nd	+	nd	[108]
**111**	Raffinose	+	nd	nd	nd	nd	nd	nd	nd	nd	nd	nd	nd	nd	nd	nd	nd	nd	nd	nd	nd	[106]
**112**	Stachyose	-	nd	nd	nd	nd	nd	nd	nd	nd	nd	nd	nd	nd	nd	nd	nd	nd	nd	+	nd	[108]
**113**	Starch	+	nd	nd	nd	nd	nd	nd	nd	nd	nd	nd	nd	nd	nd	nd	nd	nd	nd	nd	nd	[106]
**114**	4-*O*-methyl glucuronic acid	+	nd	nd	nd	nd	nd	nd	nd	nd	nd	nd	nd	nd	nd	nd	nd	nd	nd	nd	nd	[43]
	**Amino Acids**																					
**115**	Alanine	nd	nd	nd	nd	nd	nd	+	nd	nd	nd	nd	nd	nd	nd	nd	nd	nd	nd	nd	nd	[110]
**116**	Aspartate	nd	nd	nd	nd	nd	nd	+	nd	nd	nd	nd	nd	nd	nd	nd	nd	nd	nd	nd	nd	[110]
**117**	Asparagine	nd	nd	nd	nd	nd	nd	+	nd	nd	nd	nd	nd	nd	nd	nd	nd	nd	nd	nd	nd	[110]
**118**	Cysteine	nd	nd	nd	nd	nd	nd	+	nd	nd	nd	nd	nd	nd	nd	nd	nd	nd	nd	nd	nd	[110]
**119**	Glutamate	nd	nd	nd	nd	nd	nd	+	nd	nd	nd	nd	nd	nd	nd	nd	nd	nd	nd	nd	nd	[110]
**120**	Glycine	nd	nd	nd	nd	nd	nd	+	nd	nd	nd	nd	nd	nd	nd	nd	nd	nd	nd	nd	nd	[110]
**121**	Glycinebetain	nd	nd	nd	nd	nd	nd	+[a]	nd	nd	nd	nd	nd	nd	+[b]	nd	nd	+[c]	nd	nd	nd	[a][110]; [b, c][109]
**122**	Histidine	nd	nd	nd	nd	nd	nd	+	nd	nd	nd	nd	nd	nd	nd	nd	nd	nd	nd	nd	nd	[110]
**123**	Leucine	nd	nd	nd	nd	nd	nd	+	nd	nd	nd	nd	nd	nd	nd	nd	nd	nd	nd	nd	nd	[110]
**124**	Lysine	nd	nd	nd	nd	nd	nd	+	nd	nd	nd	nd	nd	nd	nd	nd	nd	nd	nd	nd	nd	[110]
**125**	Methionine	nd	nd	nd	nd	nd	nd	+	nd	nd	nd	nd	nd	nd	nd	nd	nd	nd	nd	nd	nd	[110]
**126**	Phenylalanine	nd	nd	nd	nd	nd	nd	+	nd	nd	nd	nd	nd	nd	nd	nd	nd	nd	nd	nd	nd	[110]
**127**	Proline	nd	nd	nd	nd	nd	nd	+[a]	nd	nd	nd	nd	nd	nd	+[b]	nd	nd	+[c]	nd	nd	nd	[a][110]; [b, c][109]
**128**	Serotonin	nd	nd	nd	nd	nd	nd	+	nd	nd	nd	nd	nd	nd	nd	nd	nd	nd	nd	nd	nd	[110]
**129**	Threonine	nd	nd	nd	nd	nd	nd	+	nd	nd	nd	nd	nd	nd	nd	nd	nd	nd	nd	nd	nd	[110]
**130**	Tyrosine	nd	nd	nd	nd	nd	nd	+	nd	nd	nd	nd	nd	nd	nd	nd	nd	nd	nd	nd	nd	[110]
**131**	Valine	nd	nd	nd	nd	nd	nd	+	nd	nd	nd	nd	nd	nd	nd	nd	nd	nd	nd	nd	nd	[110]
	**Alcohols, Aldehydes and Ketones**																					
**132**	Pent-4-en-1-ol	nd	nd	+	nd	nd	nd	nd	nd	nd	nd	nd	nd	nd	nd	nd	nd	nd	nd	nd	nd	[57]
**133**	Butane-di-1,4-ol	nd	nd	nd	nd	+	nd	nd	nd	nd	nd	nd	nd	nd	nd	nd	nd	nd	nd	nd	nd	[65]
**134**	But-2-en-3-ol	nd	nd	+	nd	nd	nd	nd	nd	nd	nd	nd	nd	nd	nd	nd	nd	nd	nd	nd	nd	[57]
**135**	Hexanol	nd	nd	nd	nd	nd	nd	nd	nd	nd	nd	nd	nd	+	nd	nd	nd	nd	nd	nd	nd	[236]
**136**	Cyclohexanol	nd	nd	+	nd	nd	nd	nd	nd	nd	nd	nd	nd	nd	nd	nd	nd	nd	nd	nd	nd	[57]

**137**	2-Hexadecenol	nd	nd	+[a]	nd	+[b]	nd	nd	nd	nd	nd	nd	nd	nd	nd	nd	nd	nd	nd	nd	nd	[a][56]; [b][64,237]
**138**	Octenol	nd	nd	nd	nd	nd	nd	nd	nd	nd	nd	nd	nd	+	nd	nd	nd	nd	nd	nd	nd	[236]
**139**	Undecan-1-ol	nd	nd	+	nd	nd	nd	nd	nd	nd	nd	nd	nd	nd	nd	nd	nd	nd	nd	nd	nd	[56]
**140**	Undec-2-enol	nd	nd	nd	nd	nd	nd	nd	nd	nd	nd	nd	nd	nd	nd	+	nd	nd	nd	nd	nd	[59]
**141**	Dodecanol	nd	nd	+	nd	nd	nd	nd	nd	nd	nd	nd	nd	nd	nd	nd	nd	nd	nd	nd	nd	[56]
**142**	Tridecanol	nd	nd	+	nd	nd	nd	nd	nd	nd	nd	nd	nd	nd	nd	nd	nd	nd	nd	nd	nd	[56,57]
**143**	Pentadecanol	nd	nd	+	nd	nd	nd	nd	nd	nd	nd	nd	nd	nd	nd	nd	nd	nd	nd	nd	nd	[56]
**144**	Hexacosanol	+[a]	nd	nd	nd	nd	nd	nd	nd	nd	nd	nd	+[b]	nd	nd	nd	nd	nd	nd	nd	nd	[a][43]; [b][238]
**145**	Heptadecan-1-ol	nd	nd	+	nd	nd	nd	nd	nd	nd	nd	nd	nd	nd	nd	nd	nd	nd	nd	nd	nd	[56]
**146**	Octacosanol	+[a]	nd	nd	nd	nd	nd	nd	nd	nd	nd	nd	+[b]	nd	nd	nd	nd	nd	nd	nd	nd	[a][43]; [b][238]
**147**	Nonadecan-1-ol	nd	nd	+	nd	nd	nd	nd	nd	nd	nd	nd	nd	nd	nd	nd	nd	nd	nd	nd	nd	[56]
**148**	Docosanol	nd	nd	nd	nd	nd	nd	nd	nd	nd	nd	nd	+	nd	nd	nd	nd	nd	nd	nd	nd	[238]
**149**	Tetracosanol	nd	nd	nd	nd	nd	nd	nd	nd	nd	nd	nd	+	nd	nd	nd	nd	nd	nd	nd	nd	[238]
**150**	Triacontanol	+[a]	nd	nd	nd	nd	nd	nd	nd	nd	nd	nd	+[b]	nd	nd	nd	nd	nd	nd	nd	nd	[a][43]; [b][238]
**151**	Dotriacontanol	+[a]	nd	nd	nd	nd	nd	nd	nd	nd	nd	nd	+[b]	nd	nd	nd	nd	nd	nd	nd	nd	[a][43]; [b][238]
**152**	Glycerol	nd	nd	nd	nd	+[a]	nd	+[b]	nd	nd	nd	nd	nd	nd	nd	nd	nd	nd	nd	nd	nd	[a][66]; [110]
**153**	Benzenediol	nd	nd	nd	nd	+	nd	nd	nd	nd	nd	nd	nd	nd	nd	nd	nd	nd	nd	nd	nd	[64]
**154**	Inositol	nd	nd	nd	nd	nd	nd	nd	nd	nd	nd	nd	nd	nd	nd	nd	nd	+	nd	nd	nd	[109]
**155**	2-Methoxy-4-vinylphenol	nd	nd	nd	nd	+	nd	nd	nd	nd	nd	nd	nd	nd	nd	nd	nd	nd	nd	nd	nd	[64]
**156**	Pentadecanal	nd	nd	+	nd	nd	nd	nd	nd	nd	nd	nd	nd	nd	nd	nd	nd	nd	nd	nd	nd	[56]
**157**	Hexadecanal	nd	nd	nd	nd	+	nd	nd	nd	nd	nd	nd	nd	nd	nd	nd	nd	nd	nd	nd	nd	[58]
**158**	Octadecanal	nd	nd	+	nd	nd	nd	nd	nd	nd	nd	nd	nd	nd	nd	nd	nd	nd	nd	nd	nd	[56]
**159**	9-Acetoxynonadal	nd	nd	nd	nd	nd	nd	nd	nd	nd	nd	nd	nd	nd	nd	nd	nd	nd	nd	nd	nd	[59]
**160**	Hexacosanal	+	nd	nd	nd	nd	nd	nd	nd	nd	nd	nd	nd	nd	nd	nd	nd	nd	nd	nd	nd	[43]
**161**	Octacosanal	+	nd	nd	nd	nd	nd	nd	nd	nd	nd	nd	nd	nd	nd	nd	nd	nd	nd	nd	nd	[43]
**162**	Triacontanal	+	nd	nd	nd	nd	nd	nd	nd	nd	nd	nd	nd	nd	nd	nd	nd	nd	nd	nd	nd	[43]
**163**	Benzaldehyde	nd	nd	nd	nd	+	nd	nd	nd	nd	nd	nd	nd	nd	nd	nd	nd	nd	nd	nd	nd	[64]
**164**	4-Hydroxy benzaldehyde	nd	nd	nd	nd	+	nd	nd	nd	nd	nd	nd	nd	nd	nd	nd	nd	nd	nd	nd	nd	[234]

**165**	Undecanone	nd	nd	+	nd	nd	nd	nd	nd	nd	nd	nd	nd	nd	nd	nd	nd	nd	nd	nd	nd	[56]
**166**	Pentadecanone	nd	nd	+	nd	nd	nd	nd	nd	nd	nd	nd	nd	nd	nd	nd	nd	nd	nd	nd	nd	[56]
	**Triterpenoids**																					
**167**	β-Amyrenone	+	nd	nd	nd	nd	nd	nd	nd	nd	nd	nd	nd	nd	nd	nd	nd	nd	nd	nd	nd	[43]
**168**	α-Amyrenone	+	nd	nd	nd	nd	nd	nd	nd	nd	nd	nd	nd	nd	nd	nd	nd	nd	nd	nd	nd	[43]
**169**	β-Amyrin	+	nd	nd	nd	nd	nd	nd	nd	nd	nd	nd	nd	nd	nd	nd	nd	nd	nd	nd	nd	[43]
**170**	α-Amyrin	+[a]	nd	nd	nd	nd	nd	nd	nd	nd	+[b]	nd	nd	nd	nd	nd	nd	nd	nd	nd	nd	[a][43] [b][239]
**171**	β-Amyrinyl ester	+	nd	nd	nd	nd	nd	nd	nd	nd	nd	nd	nd	nd	nd	nd	nd	nd	nd	nd	nd	[43]
**172**	α-Amyrinyl ester	+	nd	nd	nd	nd	nd	nd	nd	nd	nd	nd	nd	nd	nd	nd	nd	nd	nd	nd	nd	[43]
**173**	Arundoin	nd	nd	nd	nd	+[a]	nd	nd	nd	nd	+[b]	nd	nd	nd	nd	nd	nd	nd	nd	nd	nd	[a][58]; [b][88]
**174**	Cylindrin	nd	nd	nd	nd	nd	nd	nd	nd	nd	+	nd	nd	nd	nd	nd	nd	nd	nd	nd	nd	[88]
**175**	Fernenol	nd	nd	nd	nd	nd	nd	nd	nd	nd	+	nd	nd	nd	nd	nd	nd	nd	nd	nd	nd	[88]
**176**	Friedelin	nd	nd	nd	nd	+	nd	nd	nd	nd	nd	nd	nd	nd	nd	nd	nd	nd	nd	nd	nd	[58]
**177**	Lupeol	nd	nd	+	nd	nd	nd	nd	nd	nd	nd	nd	nd	nd	nd	nd	nd	nd	nd	nd	nd	[56]
**178**	Simiarenol	nd	nd	nd	nd	nd	nd	nd	nd	nd	+	nd	nd	nd	nd	nd	nd	nd	nd	nd	nd	[88]
**179**	Squalene	nd	nd	+[a]	nd	+[b]	nd	nd	nd	nd	nd	nd	nd	nd	nd	nd	nd	nd	nd	nd	nd	[a][56]; [b][66]
**180**	Ursolic acid	+	nd	nd	nd	nd	nd	nd	nd	nd	nd	nd	nd	nd	nd	nd	nd	nd	nd	nd	nd	[233]
	**Sesquiterpenoids**																					
**181**	Aromadendrene	nd	nd	nd	nd	nd	nd	nd	nd	nd	nd	nd	nd	+	nd	nd	nd	nd	nd	nd	nd	[236]
**182**	Bisabolene	nd	nd	nd	nd	nd	nd	nd	nd	nd	nd	nd	nd	nd	nd	+	nd	nd	nd	nd	nd	[59]
**183**	Caryophyllene oxide	nd	nd	nd	nd	nd	+	nd	nd	nd	nd	nd	nd	nd	nd	nd	nd	nd	nd	nd	nd	[160]
**184**	Cylindrene	nd	nd	nd	nd	nd	nd	nd	nd	nd	+	nd	nd	nd	nd	nd	nd	nd	nd	nd	nd	[240]
**185**	Curlone	nd	nd	nd	nd	+	nd	nd	nd	nd	nd	nd	nd	nd	nd	nd	nd	nd	nd	nd	nd	[64]
**186**	β-Eudesmol	nd	nd	nd	nd	nd	+	nd	nd	nd	nd	nd	nd	nd	nd	nd	nd	nd	nd	nd	nd	[160]
**187**	Eseroline	nd	nd	nd	nd	nd	+	nd	nd	nd	nd	nd	nd	nd	nd	nd	nd	nd	nd	nd	nd	[160]
**188**	Glutinone	nd	nd	nd	nd	nd	nd	nd	nd	nd	+	nd	nd	nd	nd	nd	nd	nd	nd	nd	nd	[88]
**189**	Tumerone	nd	nd	nd	nd	+	nd	nd	nd	nd	nd	nd	nd	nd	nd	nd	nd	nd	nd	nd	nd	[64]
	**Steroids and Derivatives**																					
**190**	Androst-4-en-3-one	nd	nd	+	nd	nd	nd	nd	nd	nd	nd	nd	nd	nd	nd	nd	nd	nd	nd	nd	nd	[56]
**191**	Campesterol	+	nd	nd	nd	nd	nd	nd	nd	nd	nd	nd	nd	nd	nd	nd	nd	nd	nd	nd	nd	[43]

**192**	Campesteryl 3-β-dglucopyranoside	+	nd	nd	nd	nd	nd	nd	nd	nd	nd	nd	nd	nd	nd	nd	nd	nd	nd	nd	nd	[43]
**193**	Cycloartenone	+	nd	nd	nd	nd	nd	nd	nd	nd	nd	nd	nd	nd	nd	nd	nd	nd	nd	nd	nd	[43]
**194**	Cholest-22-ene-21-ol	nd	nd	+	nd	nd	nd	nd	nd	nd	nd	nd	nd	nd	nd	nd	nd	nd	nd	nd	nd	[56]
**195**	Ergost-5-en-3-ol	nd	nd	+	nd	nd	nd	nd	nd	nd	nd	nd	nd	nd	nd	nd	nd	nd	nd	nd	nd	[56]
**196**	β-Sitosterol	+[a]	nd	nd	nd	+[b]	+[c]	nd	nd	nd	+[d]	nd	nd	nd	nd	nd	nd	nd	nd	nd	nd	[a][43,233]; [b][58]; [c][53]; [d][239]
**197**	β-Sitosterol glucoside	+[a]	nd	nd	nd	nd	+[b]	nd	nd	nd	+[c]	nd	nd	nd	nd	nd	nd	nd	nd	nd	nd	[a][233]; [b][53]; [c][239]
**198**	7-oxo-Sitosterol	+	nd	nd	nd	nd	nd	nd	nd	nd	nd	nd	nd	nd	nd	nd	nd	nd	nd	nd	nd	[43]
**199**	Sitosteryl ester	+	nd	nd	nd	nd	nd	nd	nd	nd	nd	nd	nd	nd	nd	nd	nd	nd	nd	nd	nd	[43]
**200**	Sitosteryl 3-B-d-glucopyranoside	+	nd	nd	nd	nd	nd	nd	nd	nd	nd	nd	nd	nd	nd	nd	nd	nd	nd	nd	nd	[43]
**201**	Stigmasterol	+[a]	nd	+[b]	nd	nd	+[c]	nd	nd	nd	nd	nd	nd	nd	nd	nd	nd	nd	nd	nd	nd	[a][43]; [b][56]; [c][53]
**202**	Stigmastanol	+	nd	nd	nd	nd	nd	nd	nd	nd	nd	nd	nd	nd	nd	nd	nd	nd	nd	nd	nd	[43]
**203**	Stigmasta-3,5-dien-7-one	+[a]	nd	+[b]	nd	nd	nd	nd	nd	nd	nd	nd	nd	nd	nd	nd	nd	nd	nd	nd	nd	[a][43]; [b][56]
**204**	Stigmast-4-en-3-one	+[a]	nd	+[b]	nd	nd	nd	nd	nd	nd	nd	nd	nd	nd	nd	nd	nd	nd	nd	nd	nd	[a][43]; [b][56]
**205**	Stigmast-4-en-3,6-dione	+	nd	nd	nd	nd	nd	nd	nd	nd	nd	nd	nd	nd	nd	nd	nd	nd	nd	nd	nd	[43,233]
**206**	Stigmastane-3,6-dione	+	nd	nd	nd	nd	nd	nd	nd	nd	nd	nd	nd	nd	nd	nd	nd	nd	nd	nd	nd	[43]
**207**	Stigmast-5-en-3β,7β-diol	nd	nd	nd	nd	nd	+	nd	nd	nd	nd	nd	nd	nd	nd	nd	nd	nd	nd	nd	nd	[53]
**208**	Stigmast-5-en-3-ol	nd	nd	+	nd	nd	nd	nd	nd	nd	nd	nd	nd	nd	nd	nd	nd	nd	nd	nd	nd	[56]
**209**	Stigmasteryl 3-β-d-glucopyranoside	+	nd	nd	nd	nd	nd	nd	nd	nd	nd	nd	nd	nd	nd	nd	nd	nd	nd	nd	nd	[43]
	**Other terpenoids**																					
**210**	B-Carotene	nd	nd	nd	nd	+	nd	nd	nd	nd	nd	nd	nd	nd	nd	nd	nd	nd	nd	nd	nd	[58,241]
**211**	Camphene	nd	nd	nd	nd	nd	+	nd	nd	nd	nd	nd	nd	nd	nd	nd	nd	nd	nd	nd	nd	[160]

**212**	Limonene	nd	nd	nd	nd	nd	+	nd	nd	nd	nd	nd	nd	nd	nd	nd	nd	nd	nd	nd	nd	[160]
**213**	Menthol	nd	nd	nd	nd	+	nd	nd	nd	nd	nd	nd	nd	nd	nd	nd	nd	nd	nd	nd	nd	[64]
**214**	Naphthalene	nd	nd	+	nd	nd	nd	nd	nd	nd	nd	nd	nd	nd	nd	nd	nd	nd	nd	nd	nd	[56]
**215**	α-Pinene	nd	nd	nd	nd	nd	nd	nd	nd	nd	nd	nd	nd	+	nd	nd	nd	nd	nd	nd	nd	[236]
**216**	Phytol	nd	nd	+[a]	nd	+[b]	nd	nd	nd	nd	nd	nd	nd	nd	nd	+[c]	nd	nd	nd	nd	nd	[a][56]; [b][58,64–66]; [c][59]
	**Tocopherols**																					
**217**	Tocopherol	+[a]	nd	nd	nd	nd	nd	nd	nd	nd	nd	nd	+[b]	nd	nd	nd	nd	nd	nd	nd	nd	[a][43]; [b][238]
**218**	β-Tocopherol	+	nd	nd	nd	nd	nd	nd	nd	nd	nd	nd	nd	nd	nd	nd	nd	nd	nd	nd	nd	[43]
**219**	α-Tocopherol	+	nd	nd	nd	nd	nd	nd	nd	nd	nd	nd	nd	nd	nd	nd	nd	nd	nd	nd	nd	[43]
**220**	γ-Tocopherol	nd	nd	+	nd	nd	nd	nd	nd	nd	nd	nd	nd	nd	nd	nd	nd	nd	nd	nd	nd	[56]
	**Flavonoids**																					
**221**	Apigenin	nd	nd	nd	nd	+	nd	nd	nd	nd	nd	nd	nd	nd	nd	nd	nd	nd	nd	nd	nd	[58,82]
**222**	Apigenin-8-*C*-sophoroside	nd	nd	nd	nd	+[a]	nd	nd	+[b]	nd	nd	nd	nd	nd	nd	nd	nd	nd	nd	nd	nd	[a][242]; [b][90]
**223**	6-*C*-pentosyl-8-*C*-hexosylapigenin	nd	nd	nd	nd	+	nd	nd	nd	nd	nd	nd	nd	nd	nd	nd	nd	nd	nd	nd	nd	[242]
**224**	Catechin	nd	nd	nd	+[a]	+[b]	nd	nd	nd	nd	nd	nd	nd	nd	nd	nd	nd	nd	nd	nd	+[c]	[a][68]; [b][82,241]; [c][92]
**225**	Ellagic acid	nd	nd	nd	nd	nd	nd	nd	nd	nd	nd	nd	nd	nd	nd	nd	nd	nd	nd	nd	+	[92]
**226**	Epicatechin	nd	nd	nd	nd	nd	nd	nd	nd	nd	nd	nd	nd	nd	nd	nd	nd	nd	nd	nd	+	[92]
**227**	Epigallocatechin	nd	nd	nd	nd	nd	nd	nd	nd	nd	nd	nd	nd	nd	nd	nd	nd	nd	nd	nd	+	[92]
**228**	Isoeugenin	nd	nd	nd	nd	nd	nd	nd	nd	nd	+	nd	nd	nd	nd	nd	nd	nd	nd	nd	nd	[87]
**229**	Isoorientin	nd	nd	nd	nd	+	nd	nd	nd	nd	nd	nd	nd	nd	nd	nd	nd	nd	nd	nd	nd	[58]
**230**	Isovitexin	nd	nd	nd	nd	+	nd	nd	nd	nd	nd	nd	nd	nd	nd	nd	nd	nd	nd	nd	nd	[58]
**231**	Kaempferol	nd	nd	nd	+[a]	+[b]	+[c]	nd	nd	nd	nd	nd	nd	nd	nd	nd	nd	nd	nd	nd	+[d]	[a][68]; [b][241]; [c][53]; [d][92]
**232**	Lutein	nd	nd	nd	nd	+	nd	nd	nd	nd	nd	nd	nd	nd	nd	nd	nd	nd	nd	nd	nd	[241,242]
**233**	6-*C*-hexosyl-8-*C*-pentosylluteolin	nd	nd	nd	nd	+	nd	nd	nd	nd	nd	nd	nd	nd	nd	nd	nd	nd	nd	nd	nd	[242]
**234**	Myricetin	nd	nd	nd	nd	+	nd	nd	nd	nd	nd	nd	nd	nd	nd	nd	nd	nd	nd	nd	nd	[82,241]

**235**	Naringin	nd	nd	nd	nd	+	nd	nd	nd	nd	nd	nd	nd	nd	nd	nd	nd	nd	nd	nd	nd	[82]
**236**	Orientin 2′′-*O*-xyloside	nd	nd	nd	nd	+[a]	nd	nd	nd	nd	nd	nd	nd	nd	nd	nd	+[b]	nd	nd	nd	nd	[a][58]; [b][91]
**237**	Procyanidin	nd	nd	nd	nd	+	nd	nd	nd	nd	nd	nd	nd	nd	nd	nd	nd	nd	nd	nd	nd	[237]
**238**	Quercetin	nd	nd	nd	nd	+[a]	+[b]	nd	+[c]	nd	nd	nd	+[d]	nd	nd	+[e]	nd	nd	nd	nd	+[f]	[a][241];[b][53]; [c][90]; [d][243]; [e][195]; [f][92]
**239**	Quercetin-3-*O*-glucoside	nd	nd	nd	nd	nd	+[a]	nd	+[b]	nd	nd	nd	nd	nd	nd	nd	nd	nd	nd	nd	nd	[a][53];[b][90]
**240**	Quercetin-7-*O*-glucoside	nd	nd	nd	nd	nd	+	nd	nd	nd	nd	nd	nd	nd	nd	nd	nd	nd	nd	nd	nd	[53]
**241**	Rutin	nd	nd	nd	+[a]	+[b]	nd	nd	nd	nd	nd	nd	+[c]	nd	nd	nd	nd	nd	nd	nd	+[d]	[a][68]; [b][82,241]; [c][243]; [d][92]
**242**	Tricin	nd	nd	nd	nd	nd	+[a]	nd	+[b]	nd	nd	nd	nd	nd	nd	nd	nd	nd	nd	nd	nd	[a][53];[b][90]
**243**	Tricin-7-glucoside	nd	nd	nd	nd	nd	+[a]	nd	nd	nd	nd	nd	nd	nd	nd	nd	+[b]	nd	nd	nd	nd	[a][53];[b][91]
**244**	3,3′,4′,5′-tetrahydroxy-6,8-dimethoxy flavone	nd	nd	nd	nd	nd	nd	nd	nd	nd	nd	nd	nd	nd	nd	+	nd	nd	nd	nd	nd	[195]
**245**	5,7-dihydroxy-3′,4′,5′-trimethoxy flavone	nd	nd	nd	nd	nd	nd	nd	+	nd	nd	nd	nd	nd	nd	nd	nd	nd	nd	nd	nd	[90]
**246**	4,5,7-Trihydroxyisoflavone	nd	nd	nd	nd	+	nd	nd	nd	nd	nd	nd	nd	nd	nd	nd	nd	nd	nd	nd	nd	[237]
**247**	Dihydroxy-4′-methoxy-7-oxyglucopyronoside flavone	nd	nd	nd	nd	nd	nd	nd	nd	nd	nd	nd	nd	nd	nd	+	nd	nd	nd	nd	nd	[195]
**248**	Vitexin-2′′-*O*-xyloside	nd	nd	nd	nd	+[a]	nd	nd	nd	nd	nd	nd	nd	nd	nd	nd	+[b]	nd	nd	nd	nd	[a][58]; [b][91]
**249**	Vitexin-2′′-*O*-glucoside	nd	nd	nd	nd	nd	nd	nd	nd	nd	nd	nd	nd	nd	nd	nd	+	nd	nd	nd	nd	[91]
	**Other polyphenols**																					

**250**	Cylindol A	nd	nd	nd	nd	nd	nd	nd	nd	nd	+	nd	nd	nd	nd	nd	nd	nd	nd	nd	nd	[244]
**251**	Cylindol B	nd	nd	nd	nd	nd	nd	nd	nd	nd	+	nd	nd	nd	nd	nd	nd	nd	nd	nd	nd	[244]
**252**	Graminone A	nd	nd	nd	nd	nd	nd	nd	nd	nd	+	nd	nd	nd	nd	nd	nd	nd	nd	nd	nd	[244]
**253**	Graminone B	nd	nd	nd	nd	nd	nd	nd	nd	nd	+	nd	nd	nd	nd	nd	nd	nd	nd	nd	nd	[244]
**254**	Imperanene	nd	nd	nd	nd	nd	nd	nd	nd	nd	+	nd	nd	nd	nd	nd	nd	nd	nd	nd	nd	[244]
**255**	Scopoletin	nd	nd	nd	nd	nd	+	nd	nd	nd	nd	nd	nd	nd	nd	nd	nd	nd	nd	nd	nd	[53]
**256**	Umbelliferone	nd	nd	nd	nd	nd	+	nd	nd	nd	nd	nd	nd	nd	nd	nd	nd	nd	nd	nd	nd	[53]
**257**	4-methoxy-5-methyl coumarin-7-O-beta-D-glucopyranoside	nd	nd	nd	nd	nd	nd	nd	nd	nd	+	nd	nd	nd	nd	nd	nd	nd	nd	nd	nd	[88]
**258**	Violaxanthin	nd	nd	nd	nd	+	nd	nd	nd	nd	nd	nd	nd	nd	nd	nd	nd	nd	nd	nd	nd	[241]
**259**	Zeaxanthin	nd	nd	nd	nd	+	nd	nd	nd	nd	nd	nd	nd	nd	nd	nd	nd	nd	nd	nd	nd	[241]
	**Alkaloids**																					
**260**	Arundamine	+	nd	nd	nd	nd	nd	nd	nd	nd	nd	nd	nd	nd	nd	nd	nd	nd	nd	nd	nd	[43]
**261**	Bufotenidine	+	nd	nd	nd	nd	nd	nd	nd	nd	nd	nd	nd	nd	nd	nd	nd	nd	nd	nd	nd	[43]
**262**	Ergonovine	nd	nd	nd	nd	+	nd	nd	nd	nd	nd	nd	nd	nd	nd	nd	nd	nd	nd	nd	nd	[58]
**263**	Ergonovinine	nd	nd	nd	nd	+	nd	nd	nd	nd	nd	nd	nd	nd	nd	nd	nd	nd	nd	nd	nd	[58]
**264**	Gramine	+	nd	nd	nd	nd	nd	nd	nd	nd	nd	nd	nd	nd	nd	nd	nd	nd	nd	nd	nd	[43]
**265**	Lunamarine	nd	nd	nd	+	nd	nd	nd	nd	nd	nd	nd	nd	nd	nd	nd	nd	nd	nd	nd	nd	[68]
**266**	Pyrrolidine	nd	nd	+	nd	nd	nd	nd	nd	nd	nd	nd	nd	nd	nd	nd	nd	nd	nd	nd	nd	[56]
**267**	Pyrrolidin-2-one	nd	nd	nd	nd	+	nd	nd	nd	nd	nd	nd	nd	nd	nd	nd	nd	nd	nd	nd	nd	[64]
**268**	Pyrazolone	nd	nd	nd	nd	+	nd	nd	nd	nd	nd	nd	nd	nd	nd	nd	nd	nd	nd	nd	nd	[64]
**269**	Ribalinidine	nd	nd	nd	+	nd	nd	nd	nd	nd	nd	nd	nd	nd	nd	nd	nd	nd	nd	nd	nd	[68]
**270**	Tryptamine	+	nd	nd	nd	nd	nd	nd	nd	nd	nd	nd	nd	nd	nd	nd	nd	nd	nd	nd	nd	[43]
**271**	Tyramine	nd	nd	nd	nd	nd	nd	nd	nd	nd	nd	+	nd	nd	nd	nd	nd	nd	nd	nd	nd	[85]
	**Stilbenoids and derivatives**																					
**272**	Caraphenol C	nd	nd	nd	nd	+	nd	nd	nd	nd	nd	nd	nd	nd	nd	nd	nd	nd	nd	nd	nd	[150]
**273**	Cystibenetrimerol A	nd	nd	nd	nd	+	nd	nd	nd	nd	nd	nd	nd	nd	nd	nd	nd	nd	nd	nd	nd	[150]
**274**	Cystibenetrimerol B	nd	nd	nd	nd	+	nd	nd	nd	nd	nd	nd	nd	nd	nd	nd	nd	nd	nd	nd	nd	[150]
**275**	Laetevirenol A	nd	nd	nd	nd	+	nd	nd	nd	nd	nd	nd	nd	nd	nd	nd	nd	nd	nd	nd	nd	[150]
**276**	Leachianol G	nd	nd	nd	nd	+	nd	nd	nd	nd	nd	nd	nd	nd	nd	nd	nd	nd	nd	nd	nd	[150]

**277**	Leachianol F	nd	nd	nd	nd	+	nd	nd	nd	nd	nd	nd	nd	nd	nd	nd	nd	nd	nd	nd	nd	[150]
**278**	Pallidol	nd	nd	nd	nd	+	nd	nd	nd	nd	nd	nd	nd	nd	nd	nd	nd	nd	nd	nd	nd	[150]
**279**	Parthenostilbenin B	nd	nd	nd	nd	+	nd	nd	nd	nd	nd	nd	nd	nd	nd	nd	nd	nd	nd	nd	nd	[150]
**280**	Parthenostilbenin A	nd	nd	nd	nd	+	nd	nd	nd	nd	nd	nd	nd	nd	nd	nd	nd	nd	nd	nd	nd	[150]
**281**	Quadrangularin B	nd	nd	nd	nd	+	nd	nd	nd	nd	nd	nd	nd	nd	nd	nd	nd	nd	nd	nd	nd	[150]
**282**	Quadrangularin C	nd	nd	nd	nd	+	nd	nd	nd	nd	nd	nd	nd	nd	nd	nd	nd	nd	nd	nd	nd	[150]
**283**	Quadrangularin A	nd	nd	nd	nd	+	nd	nd	nd	nd	nd	nd	nd	nd	nd	nd	nd	nd	nd	nd	nd	[150]
**284**	Restrytisol B	nd	nd	nd	nd	+	nd	nd	nd	nd	nd	nd	nd	nd	nd	nd	nd	nd	nd	nd	nd	[150]
	**Other**																					
**285**	Benzofuran	nd	nd	nd	nd	+	nd	nd	nd	nd	nd	nd	nd	nd	nd	nd	nd	nd	nd	nd	nd	[64]
**286**	Cyanogenic hyperoside	nd	nd	nd	nd	+	nd	nd	nd	nd	nd	nd	nd	nd	nd	nd	nd	nd	nd	nd	nd	[58]
**287**	Deacetylimpecyloside	nd	nd	nd	nd	nd	nd	nd	nd	nd	+	nd	nd	nd	nd	nd	nd	nd	nd	nd	nd	[86]
**288**	Furfural	nd	nd	nd	nd	+	nd	nd	nd	nd	nd	nd	nd	nd	nd	nd	nd	nd	nd	nd	nd	[65,86,118,239]
**289**	Furfural alcohol	nd	nd	nd	nd	+	nd	nd	nd	nd	nd	nd	nd	nd	nd	nd	nd	nd	nd	nd	nd	[58]
**290**	Impecylone	nd	nd	nd	nd	nd	nd	nd	nd	nd	+	nd	nd	nd	nd	nd	nd	nd	nd	nd	nd	[86]
**291**	Impecylenolide	nd	nd	nd	nd	nd	nd	nd	nd	nd	+	nd	nd	nd	nd	nd	nd	nd	nd	nd	nd	[86]
**292**	Impecyloside	nd	nd	nd	nd	nd	nd	nd	nd	nd	+	nd	nd	nd	nd	nd	nd	nd	nd	nd	nd	[86]
**293**	Levoglucosenone	nd	nd	nd	nd	+	nd	nd	nd	nd	nd	nd	nd	nd	nd	nd	nd	nd	nd	nd	nd	[64]
**294**	Maltol	nd	nd	nd	nd	+	nd	nd	nd	nd	nd	nd	nd	nd	nd	nd	nd	nd	nd	nd	nd	[65]
**295**	Oleamide	nd	nd	nd	nd	nd	nd	nd	nd	nd	nd	nd	nd	nd	nd	+	nd	nd	nd	nd	nd	[59]
**296**	Pantolactone	nd	nd	nd	nd	+	nd	nd	nd	nd	nd	nd	nd	nd	nd	nd	nd	nd	nd	nd	nd	[64]
**297**	Pyran-4-one	nd	nd	nd	nd	+	nd	nd	nd	nd	nd	nd	nd	nd	nd	nd	nd	nd	nd	nd	nd	[64]
**298**	Seguinoside K	nd	nd	nd	nd	nd	nd	nd	nd	nd	+	nd	nd	nd	nd	nd	nd	nd	nd	nd	nd	[86]
**299**	Seguinoside K 4-methylether	nd	nd	nd	nd	nd	nd	nd	nd	nd	+	nd	nd	nd	nd	nd	nd	nd	nd	nd	nd	[86]
**300**	Triglochinin	nd	nd	nd	nd	+	nd	nd	nd	nd	nd	nd	nd	nd	nd	nd	nd	nd	nd	nd	nd	[58]
